# AI‐Assisted Engineering of Glycyrrhizic Acid/Simvastatin Nanocrystals for Multifunctional Treatment of Bacterial Osteomyelitis

**DOI:** 10.1002/advs.76694

**Published:** 2026-07-23

**Authors:** Yu Han, Yao Zhao, Chengbo Yang, Miao Niu, Jun Dai, Quanxin Ning, Kai Xiao, Jinzheng Liang, Wensheng Zhang, Liang Wang, Dan Shao, Dongsong Li

**Affiliations:** ^1^ Department of Orthopaedic Surgery Orthopaedic Center The First Hospital of Jilin University Changchun Jilin China; ^2^ State Key Laboratory of Oral Diseases and National Clinical Research Center for Oral Diseases West China Hospital of Stomatology Sichuan University Chengdu Sichuan China; ^3^ Department of Orthopedics The Third Affiliated Hospital Southern Medical University Guangzhou Guangdong China

**Keywords:** artificial intelligence, inflammation, nanocrystal, osteogenic differentiation, osteomyelitis

## Abstract

Bacterial osteomyelitis remains a formidable challenge in clinic because existing monotherapies fail to block inevitable infection, uncontrolled inflammation, and impaired bone regeneration ‌concurrently. Here, we present an AI‐assisted strategy that integrates antibacterial, anti‐inflammatory, and pro‐osteogenic activities into a single nanocrystal. Through machine learning‑assisted screening from FDA‐approved active pharmaceutical ingredients (API), we identified glycyrrhizic acid and simvastatin as a multifunctional combination capable of self‐assembling into uniform nanocrystals (SGNCs) with ultrahigh drug loading. SGNCs effectively neutralize reactive oxygen species, suppress M1 macrophage polarization, promote bactericidal effects, and reverse infection‐impaired osteogenic differentiation. Mechanistically, RNA sequencing analysis further reveals that the beneficial effects of SGNCs are associated with the inhibition of inflammatory response via cytokine‐cytokine receptor interaction pathway and the activation of bone regeneration program via the Wnt signaling pathway. As a consequence, SGNCs eradicate bacterial burden and restore bone microarchitecture with excellent biocompatibility in a rat osteomyelitis model. Our insights highlight an AI‐assisted strategy that creates a mechanism‐targeting nanomedicine solely from APIs for the efficient treatment of bacterial osteomyelitis, which currently requires multimodal management.

## Graphical abstract

Through AI‐assisted screening from FDA‐approved API to overcome the limitations of bacterial osteomyelitis treatment, glycyrrhizic acid and simvastatin are identified as a multifunctional combination capable of self‐assembling into mechanism‐targeting nanocrystals that effectively neutralize reactive oxygen species, suppress M1 macrophage polarization, promote bactericidal effects, and reverse infection‐impaired osteogenic differentiation, leading to bacterial burden eradication and bone microarchitecture restoration with excellent biocompatibility in a rat osteomyelitis model.

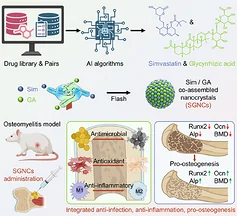

## 1 Introduction

Bacterial osteomyelitis, considered a localized bone destruction often induced by bacterial infection, is a devastating life‐threatening disease and presents significant risks of amputation and death [1, 2]. Bacterial osteomyelitis is clinically treated via surgical debridement, systemic administration of antibiotics or anti‐inflammation drugs, and pro‐osteogenic agents [3, 4]. However, excessive use of antibiotics often causes a dramatic increase in multidrug resistance [5, 6]. Beyond this challenge, overuse of anti‐inflammation agents may lead to immunosuppression that triggers secondary infection [7, 8]. The combination of antibiotics, anti‐inflammation drugs and pro‐osteogenic agents shows promise in alleviating symptoms but fails to halt disease progression, as they lack a coordinated effect [9, 10]. Therefore, there is an urgent need to develop an advanced strategy that integrates anti‐bacterial, anti‐inflammatory and pro‐osteogenic paradigms into a single therapeutic for efficient and safe treatment of bacterial osteomyelitis.

Various tailored carriers such as hydrogels, microspheres and nanoparticles, have been reported for the co‐delivery of antibiotics, immunomodulators, and regenerative factors, enabling multidimensional and comprehensive regulation of bone microenvironment [11, 12, 13]. However, most carriers are difficult to translate from bench to bedside due to the risks associated with synthetic materials and complicated manufacturing processes [14, 15]. Moreover, the low drug‐loading capacity and uncontrolled multidrug release from these carriers further restrict their therapeutic and safe outcomes [16, 17]. As an alternative, carrier‐free nanocrystals co‐assembled from two or multiple active pharmaceutical ingredients (API) without any synthetic materials have been approved for clinical use owing to their outstanding properties: (I) ultrahigh drug‐loading efficiency (even up to 100%), (II) no biosafety risks from synthetic materials; and (III) ease of large‐scale production [18, 19, 20]. Nevertheless, the design of nanocrystals is critical to ensure assembly feasibility together with integrated therapeutic benefits, which is largely governed by the selection of APIs [21, 22]. Therefore, bacterial osteomyelitis‐targeting nanocrystals should be formed from sophisticated APIs that exert anti‐bacterial, anti‐inflammatory and pro‐osteogenic effects, which still remains a significant challenge [23, 24, 25].

Although many significant technological successes have been achieved in the discovery and development of nanocrystals [26, 27], their clinical translation is hindered by specific obstacles, including time‐consuming synthesis of nanocrystals [28], an incomplete understanding of the interactions between molecular structure of API and therapeutic effects of nanocrystals [29], and challenges regarding manufacturing and the controls required for commercialization [30]. Recently, artificial intelligence (AI) approaches such as machine learning have led to exciting developments in the drug discovery, facilitating biological activity prediction and *de novo* drug design for molecular targets of interest [31, 32, 33]. Unlike traditional statistical analyses and mathematical models, AI provides deeper insights into multidimensional data, from physicochemical properties to therapeutics effects [34, 35], thus enhancing the efficiency of nanomedicine development while steering the field toward more intelligent and precise research approaches [36]. With these findings in mind, we hypothesize that AI can enable more efficient management of nanocrystal development through the sophisticated design of platform that balances assemble possibility and integrated therapeutic benefits of potent APIs — an important aspect that remains underutilized [37, 38, 39].

Here, we present an AI‐assisted nanocrystal integrating anti‐bacterial, anti‐inflammatory and pro‐osteogenic properties for efficient treatment of bacterial osteomyelitis. We first develop an AI‐framework that balances the assembly feasibility and integrated benefits of potent APIs for the rational design of bacterial osteomyelitis‐targeting nanocrystals. After experimentally screening more than 10 API combinations, we select the best‐forming co‐assembled nanocrystals (SGNCs) based on clinically approved simvastatin (Sim) and glycyrrhizic acid (GA) for further study. The former API serves as a promoter for bone regeneration [40], while the latter API is considered a multifunctional natural product with anti‐bacterial and anti‐inflammatory activities [41]. SGNCs not only alleviate oxidative stress and inflammation in vitro while exerting anti‐bacterial effect, but also enhance osteogenic differentiation of BMSCs. As a consequence, SGNCs treatment triggers initial bacterial regression in the osteomyelitis rat and produces robust osteogenic benefits with minimal side effects. Mechanistically, RNA sequencing analysis further reveals that the beneficial effects of SGNCs are associated with the inhibition of inflammatory responses via the cytokine‐cytokine receptor interaction pathway and the activation of bone regeneration programs via the Wnt signaling pathway [42]. Overall, AI‐assisted nanocrystal SGNC, featuring a high API content and the integration of multifunctional activities on anti‐infection, anti‐inflammation and pro‐osteogenesis, holds the potential to broaden the horizons of translational nanomedicine for the efficient and safe management of bacterial osteomyelitis (Scheme 1).

**SCHEME 1:**
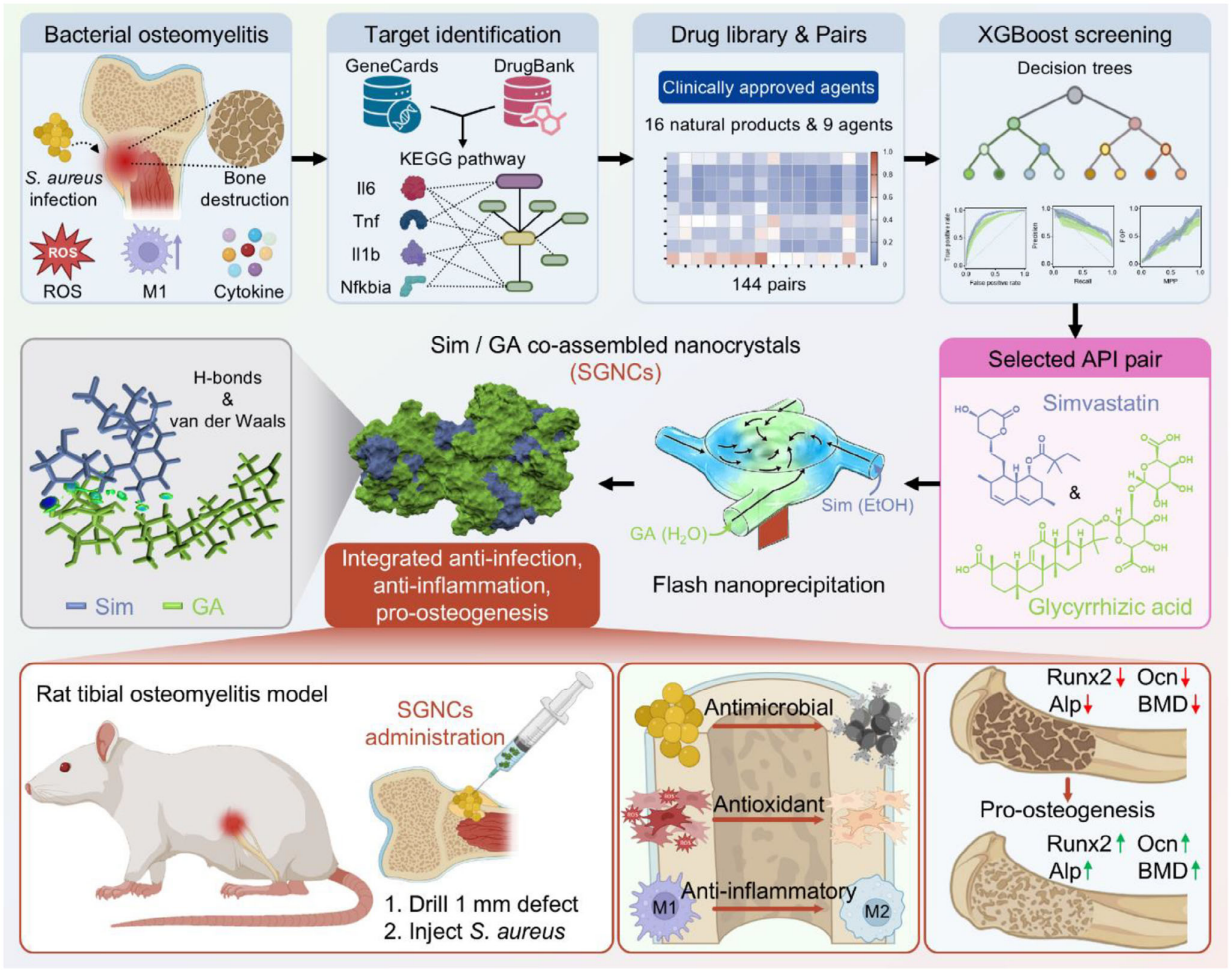
Schematic illustration of the AI‐assisted screening, self‐assembly, multifunctional therapeutic actions, and underlying mechanisms of SGNCs for the treatment of bacterial osteomyelitis.

## 2 Results and Discussion

### 2.1 AI‐Assisted Screening of Nanocrystal Compromising Anti‐Bacterial, Anti‐Inflammatory, and Pro‐Osteogenic APIs

To rationally explore API‐based therapeutics capable of addressing the interconnected pathological axes of bacterial osteomyelitis, we performed systematic target identification using an integrated multi‐database approach (Figure 1a). KEGG pathway enrichment of disease‐associated genes revealed two dominant functional clusters (Figure 1b): innate immune hyperactivation, centered on Toll‐like receptor signaling and NF‐κB activation; and bone tissue destruction, anchored by osteoclast differentiation [43]. To prioritize druggable nodes within the immune cluster, proteins were ranked by pathway contribution score and PPI network degree centrality, identifying Il6, Nfkbia, Il1b, Tnf, and Nfkb1 as the top five master regulators (Figure 1c) [44]. CTD database queries restricted to FDA‐approved natural products yielded 16 immunomodulatory compounds targeting these nodes (Figure S1) [45]; an independent curation of FDA‐approved agents with documented pro‐osteogenic activity identified a further 9 candidates (Figure S2). Pairwise combination of these two libraries generated 144 unique binary drug combinations as the input space for subsequent nanocrystal screening.

**FIGURE 1:**
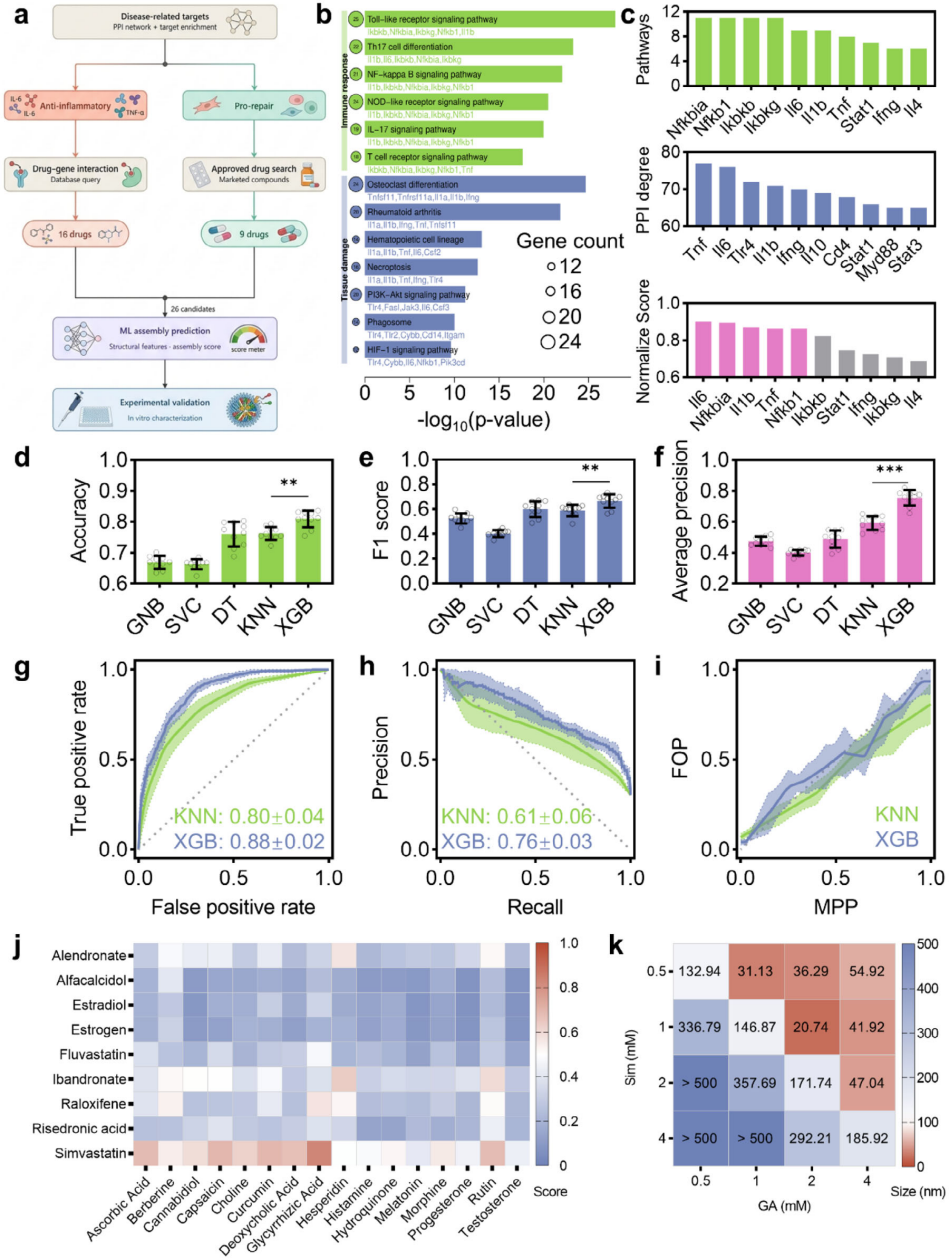
Machine learning‐guided design of nanocrystals for multifunctional intervention in bacterial osteomyelitis. (a), Schematic of nanocrystals screening workflow. (b), KEGG pathway enrichment of disease‐associated targets. (c), Top 10 genes in inflammation‐related pathways ranked by occurrence frequency (top), PPI network connectivity (middle), and normalized average (bottom). (d–f), Performance of machine learning models evaluated by 10‑fold cross‑validation: accuracy (d), F1 score (e), and average precision (f). g‐i, ROC curves (g), precision‑recall curves (h), and calibration curves (i) of XGBoost and KNN models. j, Predicted nanocrystals formation probability for all 144 drug pairs. k, Particle size distributions of the Sim‐GA combination at different molar ratios. Data are shown as mean ± SD (n = 10 for d‐f; n = 3 for k). ^**^ *p* < 0.01, ^***^ *p* < 0.001.

To efficiently prioritize nanocrystal‐forming combinations from the 144‐API library, we developed a supervised machine learning framework trained on a curated dataset of 200 experimentally validated API pairs, comprising 83 entries retrieved from the literature and 117 additional API pairs constructed experimentally in this work. Molecular descriptors for each compound were computed using RDKit (Open‐source cheminformatics software. https://www.rdkit.org), and pairwise descriptor concatenation was used as model input. Comparative evaluation across multiple classifiers under 10‐fold cross‐validation demonstrated that XGBoost achieved superior predictive performance, attaining an accuracy of 0.81 ± 0.027, alongside F1‐score, average precision, area under the ROC curve (AUC‐ROC), and area under the precision‐recall curve (AUC‐PR) that consistently outperformed K‐nearest neighbors and other benchmark models (Figure 1d–h and Figures S3–S5) [46, 47, 48]. Calibration curve analysis further confirmed that the predicted probabilities were well‐aligned with empirical assembly outcomes (Figure 1i and Figure S6). To assess external validity, we compiled an independent set of 89 literature‐reported nanoassembly systems that were absent from the training dataset; the model correctly classified 65 combinations as positive (recall = 73.0%), supporting its ability to generalize to previously unseen API pairs (Figure S7). SHAP analysis identified molecular topology‐, electronic‐state‐, and polarizability‐related descriptors as the dominant contributors to model predictions, indicating that successful nanoassembly formation is strongly influenced by molecular packing behavior and surface electronic properties (Figure S8).

Prospective application of the validated XGBoost model to all 144 API combinations assigned simvastatin (Sim) with glycyrrhizic acid (GA) the highest co‐assembly probability score, nominating it as the lead formulation candidate (Figure 1j). Experimental optimization of the Sim‐to‐GA molar feed ratio confirmed that a ratio of 1:2 yielded nanocrystals with the most favorable particle size distribution and polydispersity index (Figure 1k); this assembly condition was therefore adopted for all subsequent studies. Collectively, we employed mechanism‐guided AI framework to identify glycyrrhizic acid and simvastatin as a integrated combination capable of self‐assembling into nanocrystals with anti‐bacterial, anti‐inflammatory, and pro‐osteogenic performance.

### 2.2 Preparation and Characterization of SGNCs

Sim/GA co‐assembled nanocrystals (SGNCs) were fabricated using flash nanoprecipitation, a turbulent mixing‐based bottom‐up approach that enables scalable production of nanoparticles with controlled size distribution [49]. Transmission electron microscopy revealed that SGNCs exhibited uniform spherical morphology with an average diameter of approximately 17 nm, and high‐resolution imaging showed well‐resolved lattice fringes with crystalline nanostructure (Figure 2a, b). Dynamic light scattering confirmed a narrow size distribution with hydrodynamic diameter of 20 nm (Figure 2c). The zeta potential of −28.5 mV, attributed to surface‐exposed carboxyl groups of GA, further enhanced colloidal stability while potentially facilitating cellular internalization.

**FIGURE 2:**
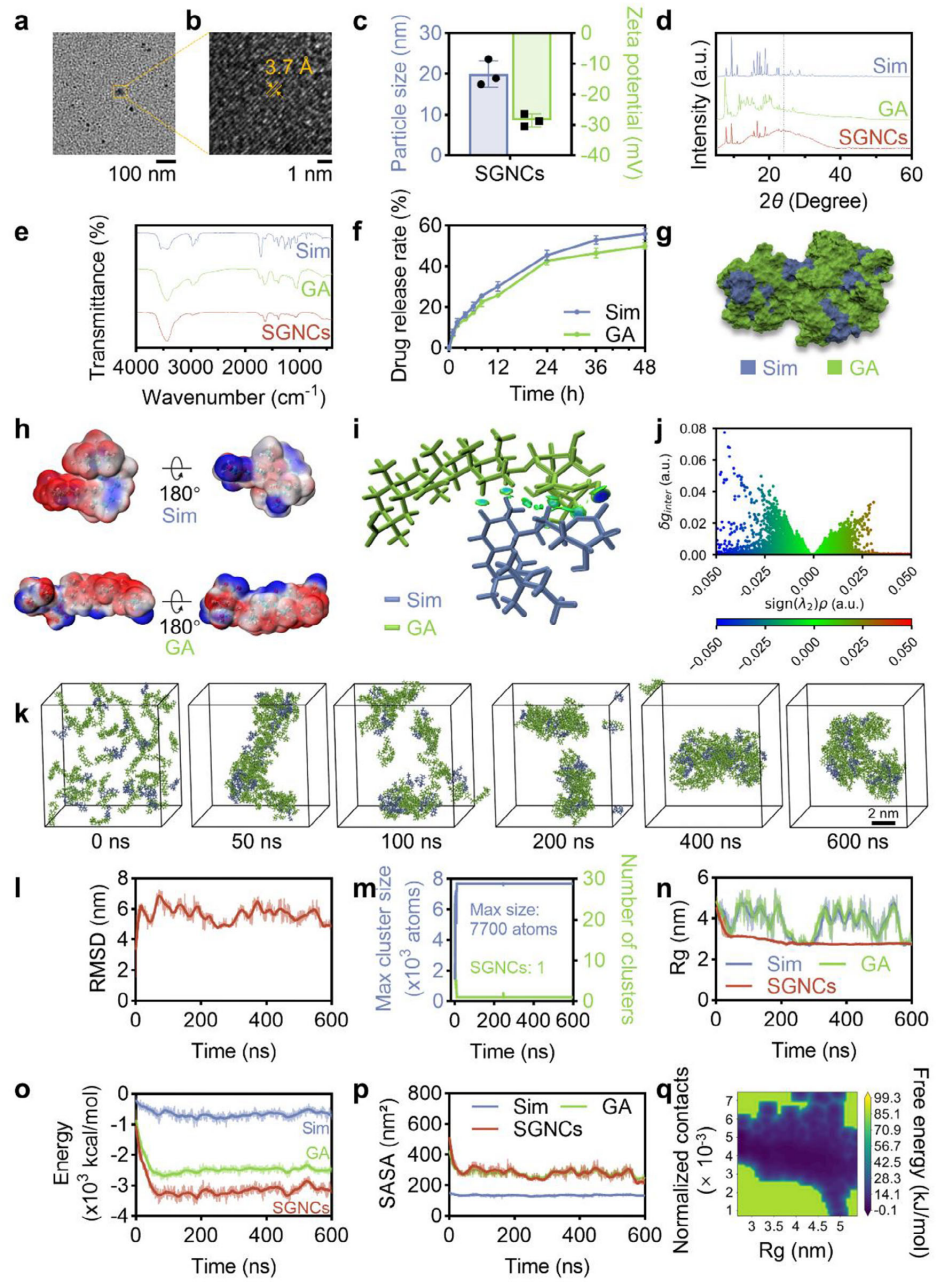
Structural characterization and molecular simulation of SGNCs. (a,b), TEM images of SGNCs (a) and corresponding lattice fringes (b). (c), Hydrodynamic size distribution and zeta potential measured by DLS. d‐e, XRD patterns (d) and FT‑IR spectra (e) of SGNCs compared with individual Sim and GA. (f), In vitro drug release profile of SGNCs under physiological conditions. (g), Schematic illustration of SGNCs assembly. (h), Electrostatic potential maps of Sim (top) and GA (bottom). (i,j), IGMH analysis of intermolecular interactions: 3D isosurfaces (i) and δg vs. sign(λ_2_)ρ scatter plot (j). (k), Representative MD snapshots at different time points. (l), RMSD of the system during simulation. (m), Time‑dependent changes in cluster number and size. (n), Radius of gyration of SGNCs, free Sim, and free GA. (o), LJ and Coulomb energy contributions during simulation. (p), SASA of the system. (q), Free energy surface of assembled SGNCs. Data are shown as mean ± SD (n = 3 for c, f).

X‐ray diffraction analysis demonstrated that SGNCs displayed a distinct diffraction pattern with new peaks emerging at 24°, while characteristic peaks from both parent compounds diminished (Figure 2d), suggesting the formation of a new crystalline phase through molecular co‐assembly rather than simple physical mixing [50]. Consistently with these findings, Fourier‐transform infrared spectroscopy further revealed that the characteristic O─H stretching band of GA at 3430 cm^−1^ shifted to 3437 cm^−1^ with pronounced broadening, while the C═O stretching peaks of GA at 1721 and 1643 cm^−1^ merged into a single broadened band at 1632 cm^−1^. Meanwhile, the lactone C═O stretching peak of Sim at 1703 cm^−1^ disappeared in the SGNCs spectrum, indicating hydrogen bonding interactions between the two assembled APIs (Figure 2e) [51]. Next, the in vitro release profiles showed sustained and closely matched release of both Sim and GA over 48 h without an initial burst (Figure 2f), which may be advantageous for achieving orchestrated therapeutics. The colloidal stability of SGNCs was further evaluated under physiological conditions and during storage. SGNCs remained stable in 10% serum at 37 °C for 7 days and as a lyophilized powder at 4°C for 180 days, with no significant changes in particle size or polydispersity index (Figure S9).

To gain molecular‐level insight into the driving forces underlying SGNCs formation (Figure 2g), we integrated quantum chemical calculations with all‐atom molecular dynamics (MD) simulations. Electrostatic potential (ESP) maps of Sim and GA revealed complementary charge distribution patterns across their molecular surfaces (Figure 2h), and independent gradient model based on Hirshfeld partition (IGMH) analysis identified multiple intermolecular interaction sites, predominantly hydrogen bonds and van der Waals contacts (Figure 2i,j) [52, 53]. MD simulations revealed that Sim and GA exhibited pronounced aggregation tendency within 50 ns, progressing toward a relatively stable cluster configuration over the full simulation trajectory (Figure 2k–n). Notably, despite comprising the minority component of the assembly, Sim was uniformly dispersed throughout the cluster with no preferential segregation to either the surface or the interior, indicating that Sim is homogeneously intercalated among GA rather than forming a discrete nucleating core. Pairwise interaction energy decomposition revealed that van der Waals forces constituted the dominant inter‐molecular contribution, with a minor but consistent hydrogen‐bonding component (Figure 2o and Figure S10). Complementary analysis of the solvent‐accessible surface area (SASA) evolution showed a progressive reduction over the simulation timescale. Furthermore, mapping of the hydrophobicity distribution across both molecules indicated that hydrophobic interactions serve as an additional critical driving force for co‐assembly during flash nanocomplexation (FNC)‐mediated fabrication (Figure 2p and Figure S11). Free energy surface (FES) analysis further corroborated the thermodynamic favorability of the assembled state, identifying a well‐defined global energy minimum corresponding to the stable SGNCs cluster conformation (Figure 2q).

### 2.3 In Vitro Antioxidant, Anti‐Inflammatory, and Antimicrobial Effects of SGNCs

Cellular uptake studies in RAW 264.7 macrophages revealed markedly enhanced internalization of SGNCs compared to free Sim, with extensive colocalization with lysosome, indicating preferential lysosomal accumulation (Figure 3a and Figure S12). Cytotoxicity assessment in BMSCs confirmed the good biocompatibility of SGNCs, with cell viability remaining >90% even at concentrations up to 50 µg/mL (Figure 3b). Taken together, these findings indicated the successful preparation of Sim/GA co‐assembled nanocrystals featuring uniform structure, controlled release, and good biocompatibility for further anti‐bacterial, anti‐inflammatory, and pro‐osteogenic applications.

**FIGURE 3:**
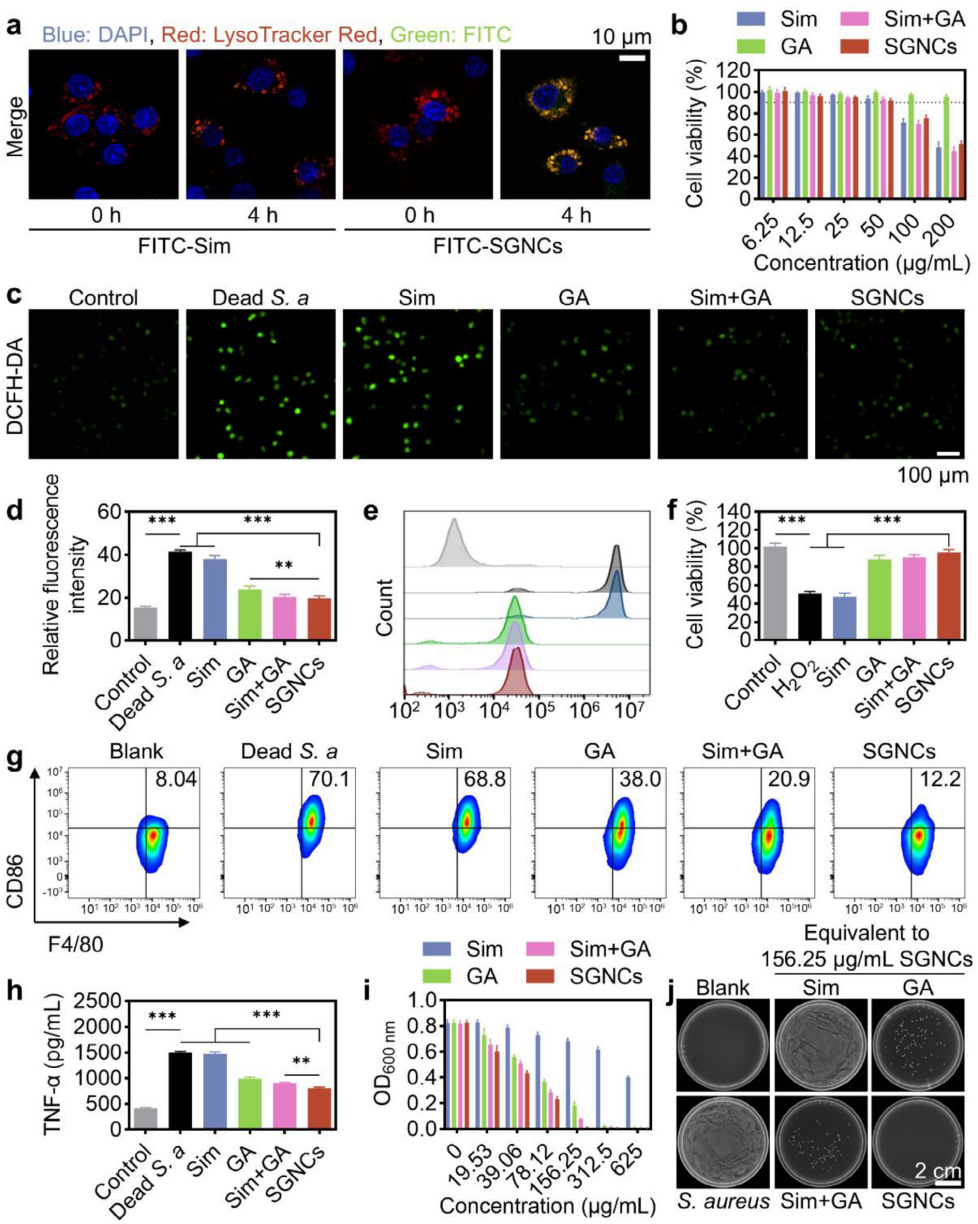
In vitro antioxidant, anti‑inflammatory and antimicrobial effects of SGNCs. (a), Confocal images of cellular uptake of free Sim and SGNCs in RAW 264.7 macrophages. Lysosomes in red, nuclei in blue, FITC‑labeled drugs in green. (b), Cytotoxicity of various formulations in BMSCs. (c,d), Representative fluorescence images (c) and quantitative analysis (d) of intracellular ROS levels. (e), Flow cytometry analysis of ROS levels. (f), Cell viability under H_2_O_2_‑induced oxidative stress. (g), Flow cytometry analysis of M1 macrophage polarization (CD86^+^ F4/80^+^). (h), TNF‑α secretion measured by ELISA. (i,j), MIC (i) and LB agar plates for MBC assays (j) against *S. aureus*. Data are shown as mean ± SD (n = 3). ^**^ *p* < 0.01, ^***^ *p* < 0.001.

Then, we evaluated the cytoprotective effects of SGNCs against bacteria‐induced oxidative stress. RAW 264.7 macrophages were exposed to heat‐inactivated *S. aureus* and treated with various formulations, with untreated cells serving as the control. Using DCFH‐DA staining, we found that inactivated bacteria robustly increased intracellular ROS levels compared to controls (Figure 3c, d). Sim treatment marginally reduced ROS fluorescence, whereas GA substantially attenuated the oxidative burst. The Sim+GA physical mixture effectively suppressed ROS production, whereas SGNCs exhibited the most pronounced antioxidant effect (Figure 3e and Figure S13). In an H_2_O_2_‐induced oxidative stress model established at 100 µM, SGNCs consistently provided superior cytoprotection compared to free GA or the physical mixture (Figure 3f).

Next, we assessed the anti‐inflammatory effects of SGNCs on bacteria‐stimulated macrophage polarization. Flow cytometric analysis revealed that inactivated *S. aureus* robustly induced M1 polarization in untreated cells, which was minimally affected by Sim treatment (Figure 3g). In contrast, SGNCs significantly reduced the proportion of M1 macrophages. These anti‐inflammatory effects of SGNCs were accompanied by the markedly suppressed TNF‐α secretion, compared to bacteria‐stimulated group (Figure 3h). Collectively, SGNCs outperformed other formulations in terms of the anti‐inflammation effects on macrophages.

Finally, antibacterial assays revealed that Sim alone lacked activity (MIC > 125 µg/mL) against live *S. aureus*, while GA showed moderate effects (MIC 250 µg/mL, MBC 250 µg/mL). The physical mixture exhibited MIC 156.25 µg/mL and MBC 312.5 µg/mL. Remarkably, SGNCs displayed enhanced potency with MIC 156.25 µg/mL and a reduced MBC of 156.25 µg/mL, indicating a transition from bacterial growth inhibition to killing (Figure 3i,j and Figure S14). In addition, SGNCs significantly inhibited *S. aureus* biofilm formation, reducing biofilm biomass to approximately 12% of the untreated group, as determined by crystal violet staining (Figure S15). In contrast, free Sim, GA, and their physical mixture showed negligible biofilm inhibitory activity. Collectively, these results demonstrated that SGNCs integrated potent antioxidant, anti‐inflammatory and antibacterial properties into a single nanotherapeutic platform, which may confer cooperative advantages under infectious and inflammatory conditions.

### 2.4 In Vitro Osteogenic Effect of SGNCs

Beyond the antioxidant, anti‐inflammatory and antimicrobial evaluation, we evaluated whether SGNCs could rescue bacteria‐impaired osteogenic differentiation. Bone marrow mesenchymal stem cells (BMSCs) were exposed to heat‐inactivated *S. aureus* and treated with various formulations for 14 days, with untreated BMSCs serving as the control [54]. Alkaline phosphatase (ALP) staining revealed that inactivated *S. aureus* profoundly suppressed early osteoblast differentiation (Figure 4a). Sim treatment partially restored ALP activity, whereas GA alone showed negligible effect. Notably, both the Sim+GA physical mixture and SGNCs enhanced ALP staining, whereas SGNCs exhibited the strongest intensity. Quantitative analysis further confirmed that SGNCs significantly increased ALP activity compared to all other treated groups (Figure 4b). Consistently, Alizarin Red S (ARS) staining for mineralized nodule formation showed that SGNCs most effectively reversed bacteria‐induced inhibition of matrix mineralization, followed by the physical mixture and Sim alone, while GA‐treated groups displayed minimal mineralization comparable to that of the model group (Figure 4a, c).

**FIGURE 4:**
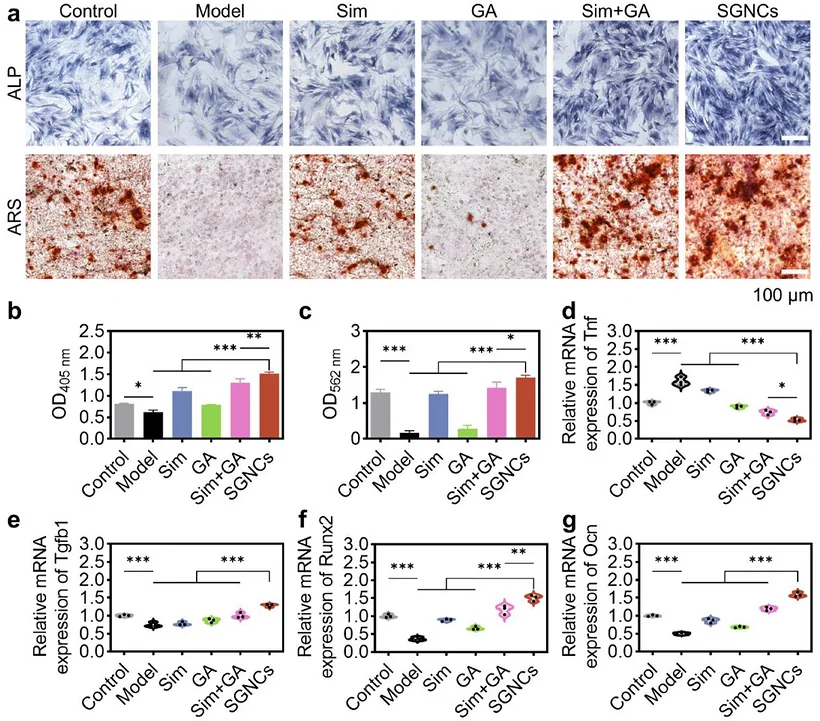
In vitro osteogenic effects of SGNCs under bacteria‑stimulated conditions. (a), Representative images of ALP staining (top) and ARS staining (bottom) in BMSCs. b‐c, Quantitative analysis of ALP activity (b) and ARS‑stained mineralized nodules (c). d‐g, Relative mRNA expression of Tnf (d), Tgfb1 (e), Runx2 (f), and Ocn (g). Data are shown as mean ± SD (n = 3). ^*^ *p* < 0.05, ^**^ *p* < 0.01, ^***^ *p* < 0.001.

To characterize the osteoinductive capacity, we next examined the expression of osteogenic and inflammatory markers by quantitative real‐time PCR. As expected, SGNCs significantly downregulated Tnf expression (Figure 4d) while upregulating Tgfb1 (Figure 4e), reflecting modulation of the inflammatory microenvironment [42]. Moreover, SGNCs significantly upregulated the expression of early transcription factor Runx2 (Figure 4f) and downstream marker Ocn (Figure 4g) compared to all other bacteria‐exposed groups [55]. The Sim+GA mixture also upregulated these osteogenic markers but to a lesser extent, whereas Sim alone showed moderate effects and GA alone was ineffective. Collectively, these results demonstrated that SGNCs markedly rescued bacteria‐impaired osteoblast differentiation and matrix mineralization through coordinated suppression of inflammation and upregulation of osteogenic programs, highlighting their therapeutic advantage for infection‐challenged bone defects.

### 2.5 The Osteogenic and Antibacterial Activities of SGNCs In Vivo

Encouraged by the anti‐bacterial, anti‐inflammatory, and pro‐osteogenic advantages of in vitro, we evaluated the protective efficacy of SGNCs using a rat tibial osteomyelitis model established by *S. aureus* inoculation at a cortical defect site (Figure S16) [56]. Four weeks post‐treatment, micro‐computed tomography (µCT) analysis with three‐dimensional reconstruction together revealed that *S. aureus* challenge induced severe bone destruction, characterized by extensive osteolysis and cortical discontinuity (Figure 5a) [57]. Quantitative analysis of bone microarchitecture demonstrated that SGNCs treatment remarkably restored bone volume fraction (BV/TV) to 22.62%, approaching the level of healthy controls (24.34%), whereas Sim (5.72%) and GA (10.29%) monotherapies showed only partial recovery (Figure 5b). Consistently, bone mineral density (BMD) in the SGNCs group reached 0.1606 g/cm^3^, which was significantly higher than that in the Sim (0.0554 g/cm^3^) and GA (0.0615g/cm^3^) groups, and comparable to that in the healthy controls (0.1743 g/cm^3^) (Figure 5c). Trabecular thickness (Tb.Th) followed a similar trend, with SGNCs (0.1489 mm) nearly restored to control levels (0.1579 mm) (Figure 5d). Furthermore, SGNCs treatment normalized infection‐induced trabecular separation (Tb.Sp) to values approaching those of control group (Figure S17), indicating robust restoration of bone microarchitecture. Bacterial load assessment by agar plate culture of bone homogenates further revealed that SGNCs treatment achieved complete bacterial clearance, comparable to that observed in healthy controls, while Sim and GA monotherapies and their physical mixture exhibited varying degrees of residual bacterial colonization (Figure 5e) [58].

**FIGURE 5:**
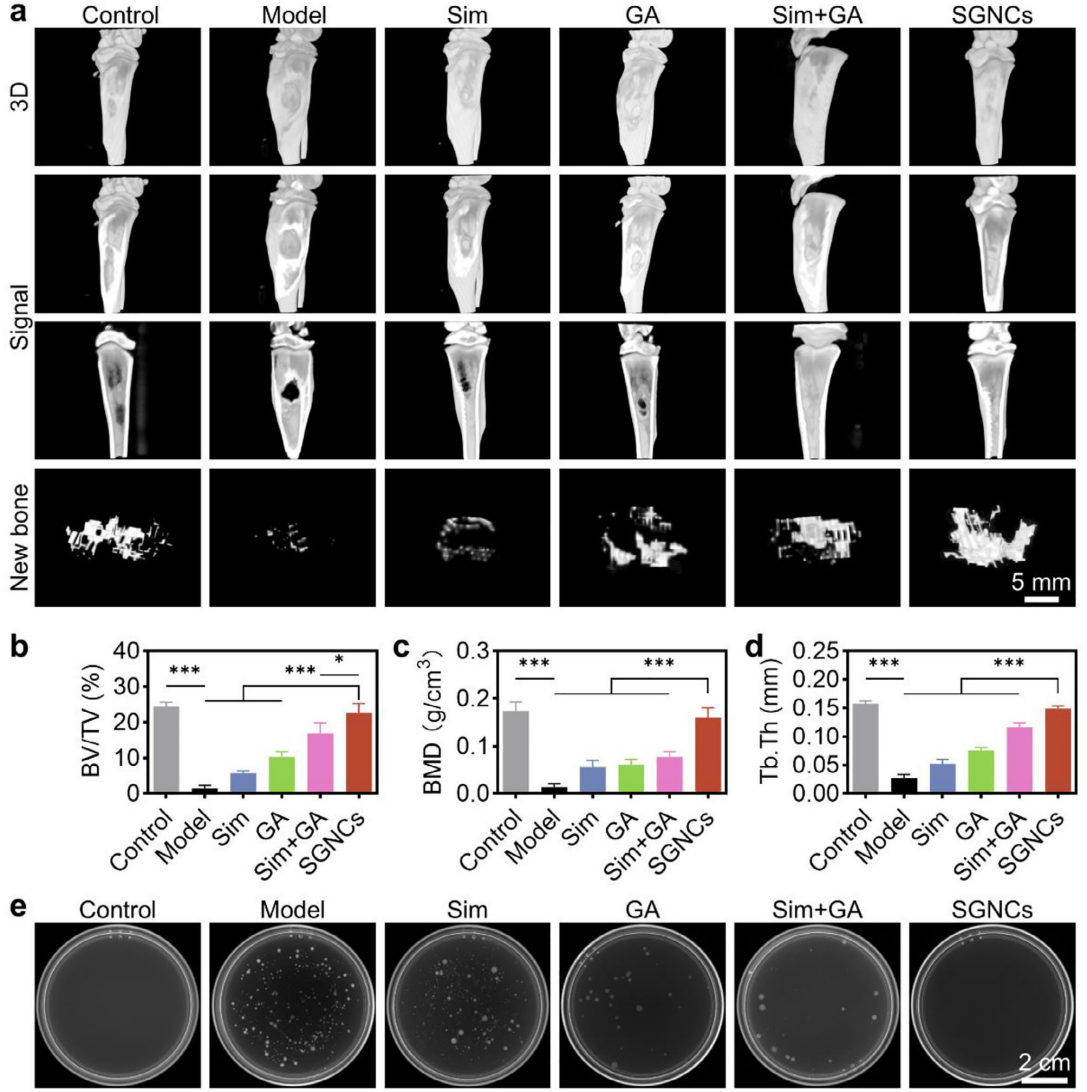
In vivo osteogenic and antibacterial efficacy of SGNCs. (a), Representative micro‑CT 3D reconstruction images of tibial defects. (b–d), Quantitative analysis of BV/TV (b), BMD (c), and Tb.Th (d). e, Bacterial load in bone homogenates assessed by agar plate culture. Data are shown as mean ± SD (n = 3). ^*^ *p* < 0.05, ^***^ *p* < 0.001.

In parallel, Hematoxylin and eosin (H&E) staining showed that infection controls exhibited dense inflammatory infiltrates and marrow destruction, which were partially reduced by Sim or GA and further ameliorated by the physical mixture. Notably, SGNCs treatment nearly completely resolved inflammation and preserved normal marrow architecture (Figure 6a). Consistently, Masson's trichrome staining revealed minimal collagen deposition in model, Sim and GA group, moderate new bone formation in physical mixture groups, and extensive bone matrix remodeling in SGNCs‐treated rat (Figure 6b and Figure S18). Immunohistochemical analysis of macrophage polarization further revealed that the iNOS‐positive M1 macrophages after infection were partially reduced by monotherapies [59]. In contrast, SGNCs treatment not only suppressed iNOS expression but also promoted CD206‐positive M2 macrophages, indicating a robust shift from pro‐inflammatory to pro‐regenerative phenotype that surpassed all other treatment groups (Figure 6c and Figure S19). Immunofluorescence staining further demonstrated that SGNCs markedly enhanced RUNX2 and OCN expression compared to other treatment groups, indicating robust osteoblast activation (Figure 6d and Figures S20 and S21) [55]. Collectively, these findings demonstrated that SGNCs profoundly eradicated infection, restored bone microarchitecture, and promoted osteogenic regeneration through the sophisticated modulation of the infectious microenvironment.

**FIGURE 6:**
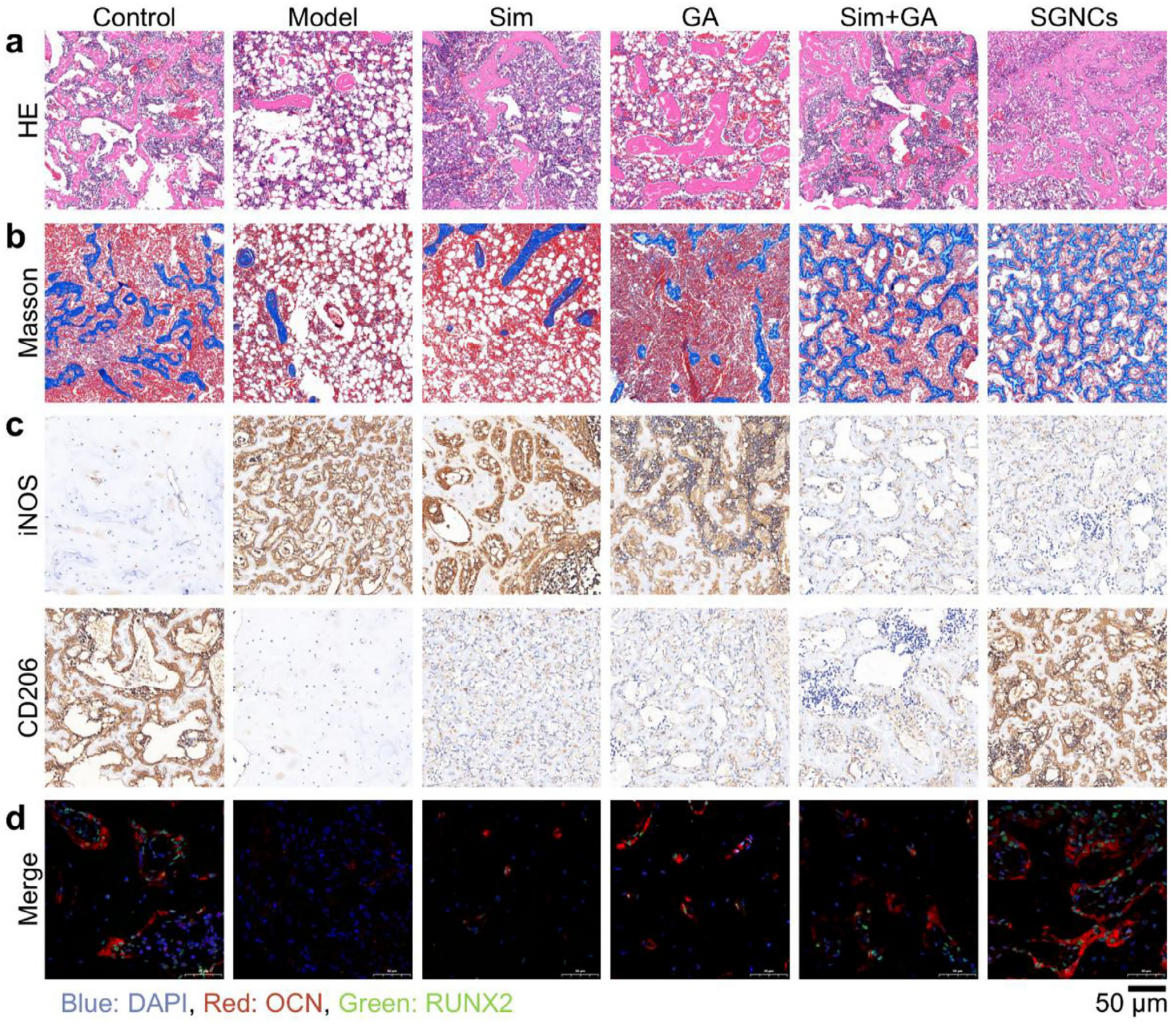
Histological and immunostaining analysis of bone tissues. (a), H&E staining of tibial sections. (b), Masson's trichrome staining. (c), Immunohistochemical staining of iNOS and CD206. (d), Immunofluorescence staining of RUNX2 (green) and OCN (red) with DAPI (blue). Scale bar, 50 µm.

### 2.6 RNA Sequencing Analysis of Protective Effects of SGNCs

To dissect the molecular basis of SGNCs efficacy, we profiled bone marrow transcriptomes from all six experimental groups [60]. Principal component analysis (PCA) revealed distinct transcriptional profiles among the groups, with SGNCs‐treated samples clustering most closely to those of control rats, whereas model and monotherapy groups formed separate clusters (Figure 7a). These results suggested a progressive restoration of the bone microenvironment transcriptome upon SGNCs treatment. Differential expression analysis identified 350 up‐regulated and 458 down‐regulated genes in SGNCs‐treated group compared to model group (|log_2_FC| > 1, padj < 0.05) (Figure 7b). Differential expression analysis (SGNCs vs model) identified that SGNCs significantly downregulated pro‐inflammatory (Il6, Cxcl16), osteoclast‐related (Acp5, Csf1r), and pathway modulators (Sfrp2, Frzb, Ctnnbip1, Smad7, Bambi). Conversely, SGNCs upregulated anti‐inflammatory mediators (Mrc1, Nfkbia, Il10), osteogenic master regulators (Runx2, Bmp2), matrix proteins (Ibsp, Spp1), and angiogenic factors (Angpt1, Vegfd) (Figure 7c).

**FIGURE 7:**
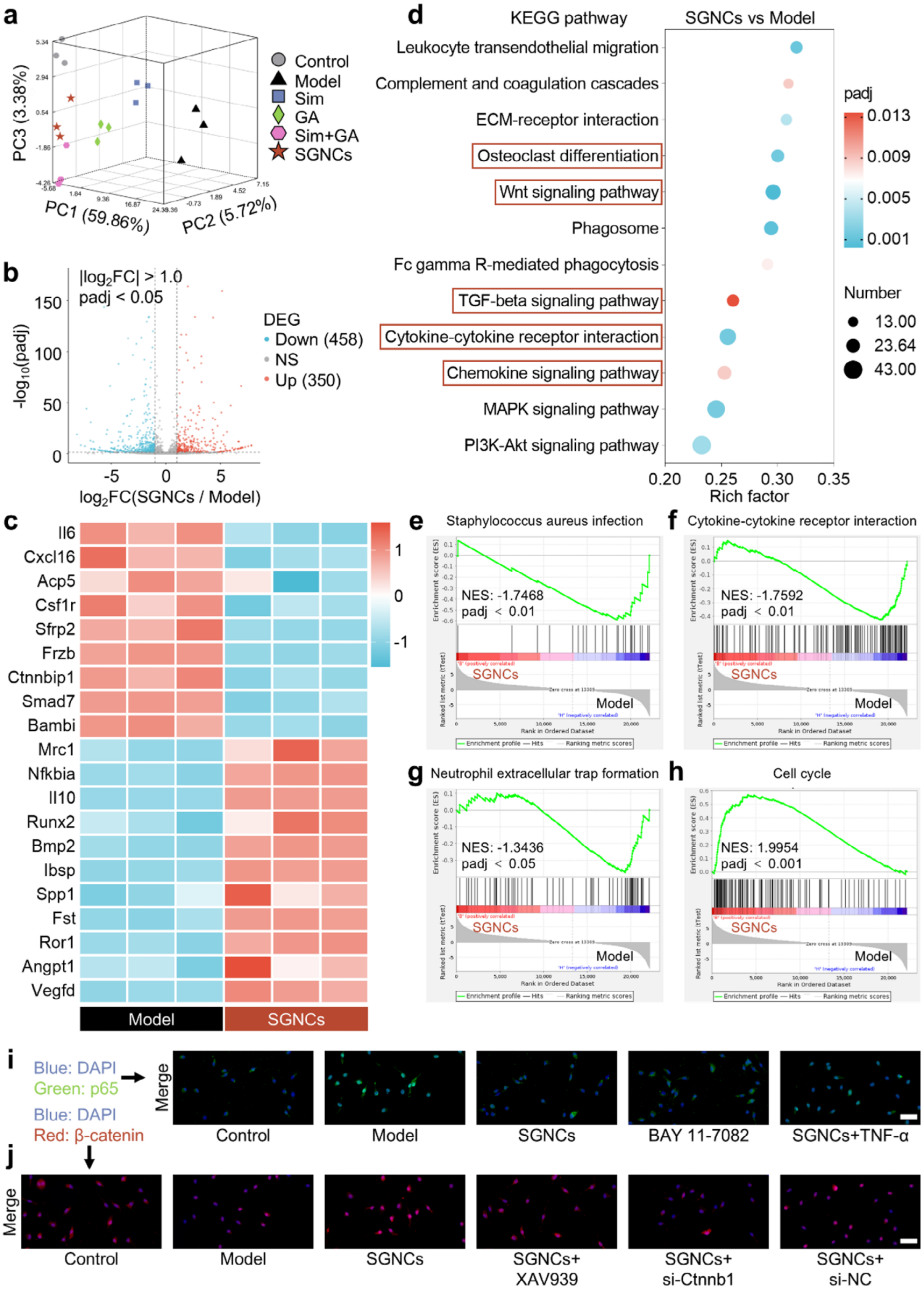
RNA sequencing analysis and immunofluorescence validation of therapeutic mechanisms. (a), PCA of transcriptomic profiles from all six groups. (b), Volcano plot of DEGs between SGNCs and model groups (|log_2_FC| > 1, padj < 0.05). (c), Heatmap of selected DEGs involved in inflammation, and osteogenesis. (d), KEGG pathway enrichment analysis. (e–h), GSEA of Staphylococcus aureus infection (e), cytokine‑cytokine receptor interaction (f), neutrophil extracellular trap formation (g), and cell cycle (h) pathways. (i), Immunofluorescence staining of p65 (green) and DAPI (blue). (j), Immunofluorescence staining of β‐catenin (red) and DAPI (blue). Scale bar, 20 µm.

KEGG pathway enrichment analysis revealed that SGNCs significantly modulated multiple signaling pathways that associated with inflammation and bone metabolism, including Osteoclast differentiation, Wnt, TGF‐beta, Cytokine‐cytokine receptor interaction, and Chemokine signaling (Figure 7d) [61]. Gene set enrichment analysis (GSEA) further substantiated these findings [62]. SGNCs treatment markedly suppressed gene sets associated with Staphylococcus aureus infection (Figure 7e) and cytokine‐cytokine receptor interaction (Figure 7f), indicating attenuated inflammatory signaling. Consistently, the neutrophil extracellular trap formation pathway was significantly downregulated (Figure 7g), suggesting reduced innate immune activation [63]. In contrast, cell cycle‐related gene sets were positively enriched in SGNCs‐treated samples (Figure 7h), pointing to enhanced cellular proliferative capacity. To experimentally validate the pathways identified by RNA‑seq, we performed immunofluorescence staining for key proteins in heat‐inactivated *S. aureus*‐stimulated BMSCs. As shown in Figure 7i and Figure S22, SGNCs treatment markedly reduced p65 fluorescence intensity compared to the model group, an effect comparable to that of the NF‐κB inhibitor BAY 11‐7082. The addition of TNF‑α partially reversed the SGNCs‐induced p65 reduction, confirming that SGNCs inhibit NF‑κB activation. For the Wnt/β‐catenin pathway, SGNCs significantly increased β‑catenin expression (Figure 7j and Figure S23). The Wnt inhibitor XAV939 and β‑catenin knockdown (si‑Ctnnb1) both attenuated this effect, whereas si‐NC had no impact, demonstrating that SGNCs activate Wnt/β‑catenin signaling. Taken together, these findings demonstrated that SGNCs exerted orchestrated protective effects through concurrent suppression of inflammatory pathways and activation of bone regeneration programs.

### 2.7 In Vivo Biosafety, Retention and Tissue Distribution of SGNCs

Finally, we evaluated the biosafety profiles of SGNCs during the four‐week treatment period. Body weight changes were analyzed by two‑way repeated‑measures ANOVA with Tukey's post hoc test. The analysis revealed a significant treatment × time interaction (p < 0.0001) and a significant main effect of treatment (p = 0.0012). Post hoc comparisons showed that the model group had significantly lower body weight than the control group at weeks 1–4. In contrast, none of the treatment groups differed significantly from the control group at any time point. These results demonstrate that all treatments, including SGNCs, were well tolerated and did not affect normal weight gain (Figure 8a). Serum biochemical analysis was performed to assess potential hepatotoxicity and nephrotoxicity (Figure 8b–d and Figure S24). Infected rats showed mild elevations in liver function markers (ALT, AST) and renal function markers (BUN, CREA) compared to blank controls, suggesting a systemic inflammatory response. In this context, Sim monotherapy partially normalized these parameters, while GA treatment showed more pronounced improvement. Notably, SGNCs treatment outperformed other treatments in restoring all serum biochemical parameters. In parallel, histopathological examination of major organs (heart, liver, spleen, lung, kidney) using H&E staining revealed no apparent morphological alterations or pathological lesions in any treatment group (Figure 8e). Despite the known potential hepatotoxicity of high‐dose Sim, no signs of drug‐induced organ damage were observed in SGNCs‐treated animals.

**FIGURE 8:**
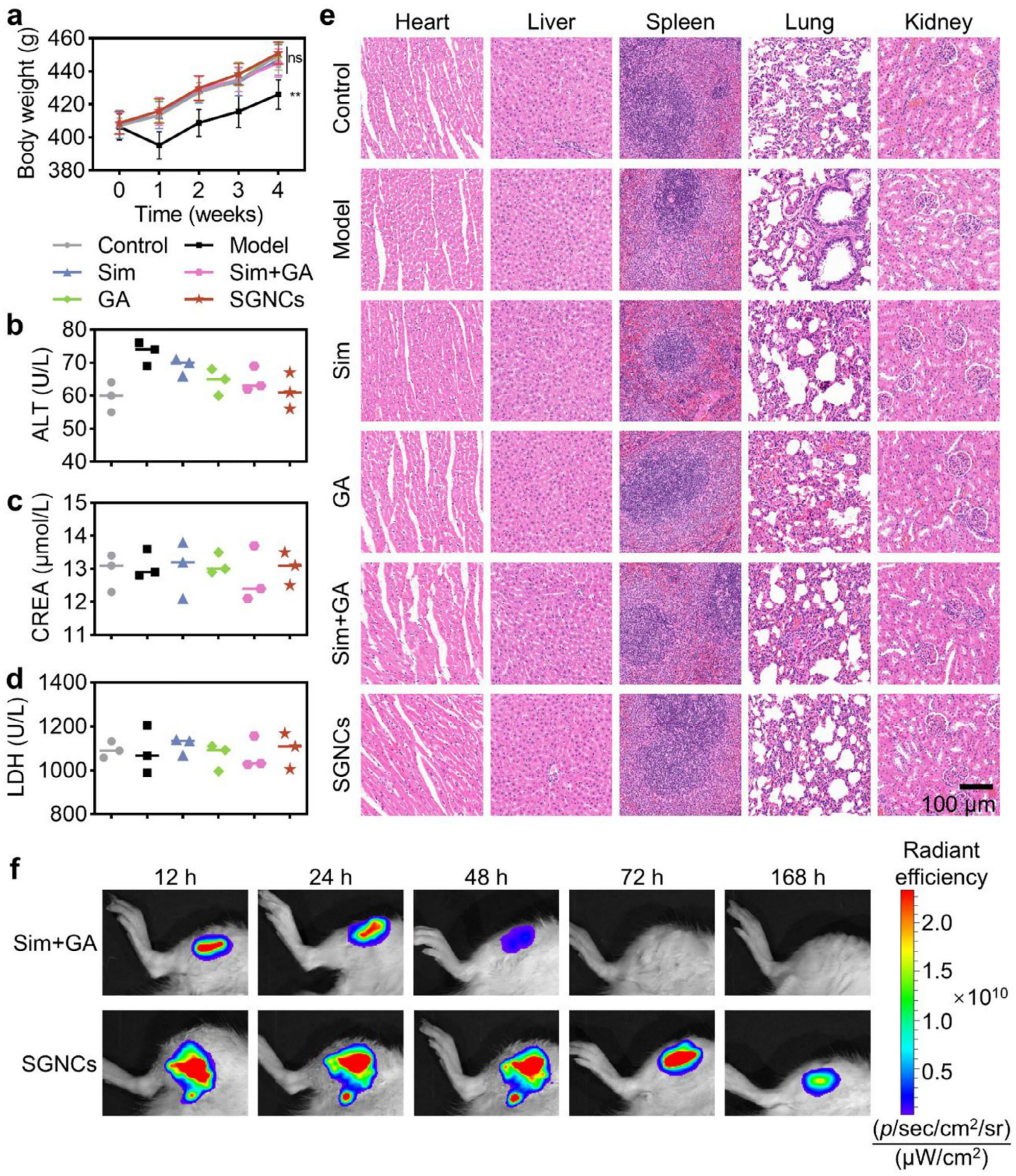
Biosafety and biodistribution profiles of SGNCs. (a), Body weight changes over 4 weeks. (b–d), Serum biochemical parameters: ALT (b), CREA (c), and LDH (d). (e), H&E staining of major organs (heart, liver, spleen, lung, kidney). Scale bar, 100 µm. (f), Representative in vivo fluorescence images of rats after local administration of Cy5‐labeled Sim+GA mixture (upper) or Cy5‐labeled SGNCs (lower) into the tibial defect. Data are shown as mean ± SD (n = 3). ^**^ *p* < 0.01.

To further evaluate the in vivo retention, bone accumulation, tissue distribution, and clearance behavior of SGNCs, we performed fluorescence imaging after local administration of Cy5‑labeled SGNCs or Cy5‑labeled Sim+GA mixture into the tibial defect. As shown in Figure 8f and Figure S25, SGNCs exhibited significantly stronger and more sustained fluorescence at the defect site than the free mixture, with detectable signal persisting for at least 168 h, whereas the mixture was almost completely cleared by 72 h. SGNCs also showed higher and more prolonged fluorescence in the tibia and liver than the mixture, reflecting slower clearance primarily via the hepatic route (Figure S26). Collectively, these results demonstrate that SGNCs exhibit favorable biodistribution and slow hepatic clearance with excellent biocompatibility, supporting their potential as a safe and long‑retaining nanotherapeutic for osteomyelitis treatment.

## 3 Conclusion

In summary, we have developed a machine learning‑guided design of nanocrystals that integrates antibacterial, anti‐inflammatory, and pro‐osteogenic APIs into a single platform for the efficient and safe management of bacterial osteomyelitis. Through AI‐assisted screening and experimental validation, we identified glycyrrhizic acid and simvastatin as a potent combination capable of self‐assembling into uniform nanocrystals with high drug loading, sustained drug release, and enhanced cellular internalization. The obtained SGNCs outperformed their simple mixtures in promoting antioxidant and anti‐inflammatory effects in macrophages, enhanced osteogenic differentiation of BMSCs under infectious conditions, and achieving complete bacterial clearance in a rat osteomyelitis model. Mechanistically, such outstanding protective effects could be explained by the concurrent suppression of the cytokine‐cytokine receptor interaction pathway and activation of bone regeneration programs, while promoting a shift from pro‐inflammatory M1 to pro‐regenerative M2 macrophage polarization. SGNCs exhibited favorable biocompatibility with no detectable systemic toxicity in the current study.

While substantial progress has been made in the development of nanocarrier‑based systems for osteomyelitis, most existing strategies rely on single antibiotic agents or require synthetic polymer carriers, often lacking intrinsic anti‑inflammatory and pro‑osteogenic activities [11]. For example, antibiotic‑loaded hydrogels or microspheres can achieve local bacterial inhibition but typically do not address the chronic inflammation or impaired bone repair that are hallmarks of the disease. In contrast, our SGNCs offer a carrier‑free platform that integrates all three functions within a single nanocrystal [64]. Furthermore, compared with conventional empirical screening of drug combinations, our AI‑assisted workflow enabled rapid prioritization of Sim/GA as a co‑assembling pair, reducing the experimental search space while maintaining high predictive accuracy [65, 66]. Thus, the true conceptual advance of this study is not the discovery of a new chemical entity, but rather the demonstration that an AI‑guided, rationally selected API pair can self‑assemble into a functional nanocrystal that simultaneously addresses the infection, inflammation, and bone loss axes of osteomyelitis.

Nevertheless, several limitations should be acknowledged. First, the antibacterial evaluation was primarily focused on *S. aureus*; activity against other pathogens remains to be tested. Second, direct comparison with clinically standard antibiotics or approved local carriers was not performed [67]. Third, the animal model represents an acute infection with immediate treatment rather than chronic, biofilm‑associated, or delayed‑diagnosis scenarios. Fourth, the biosafety assessment was limited to four weeks; long‑term toxicity, degradation kinetics, and metabolic fate require further investigation [68]. Future work will also explore imaging modalities for real‑time monitoring of biodistribution and therapeutic response, as well as dosing optimization to maximize cooperative effects and minimize systemic toxicity. Despite these challenges, our AI‑assisted nanocrystal strategy offers a promising proof‑of‑concept direction for the disease‑guided design of nanomedicines for complex conditions such as osteomyelitis that require multimodal intervention.

## 4 Experimental Section

### 4.1 Bioinformatics and Machine Learning

Disease‐associated genes for bacterial osteomyelitis were retrieved from GeneCards. KEGG pathway enrichment and protein–protein interaction (PPI) network analysis were performed using STRING. The top five targets (IL6, NFKBIA, IL1B, TNF, NFKB1) were queried in the Comparative Toxicogenomics Database (CTD) to identify FDA‐approved natural products (16 compounds). Separately, 9 FDA‐approved small molecules with documented pro‐osteogenic activity were curated from DrugBank. Pairwise combination generated 144 candidate drug pairs.

A supervised machine learning framework was trained on a dataset of 200 drug pairs (83 from literature, 117 experimentally generated). Molecular descriptors were calculated using RDKit. After 10‑fold cross‑validation of multiple classifiers (XGBoost, KNN, GNB, SVM, DT), XGBoost achieved the highest performance (accuracy 0.81 ± 0.027) and was used to screen the 144 combinations. Simvastatin (Sim) and glycyrrhizic acid (GA) received the highest co‑assembly probability score and were selected for further study.

An independent external validation set of 89 literature‐reported nanoassembly‐forming API pairs, not included in model training, was used to evaluate generalizability. Model interpretability was assessed using SHAP analysis.

### 4.2 Preparation and Characterization of SGNCs

Sim and GA were dissolved in ethanol and deionized water, respectively, at a 1:2 molar ratio and rapidly mixed using flash nanoprecipitation. The suspension was dialyzed (MWCO 3500 Da) against deionized water and lyophilized to obtain SGNCs powder. The final mass ratio of Sim to GA was approximately 1:4.

Morphology was examined by TEM (200 kV). Hydrodynamic diameter and zeta potential were measured by DLS. XRD patterns were recorded over 5–90° (2θ) at 2°/min. FT‑IR spectra were acquired. Quantum chemical calculations (ORCA 6.0, r2SCAN‑3c) and all‑atom MD simulations (GROMACS 2025.2, 600 ns) were performed to analyze the assembly mechanism.

### 4.3 In Vitro Drug Release

SGNCs (1 mg) were dispersed in PBS (pH 7.4) in dialysis bags (MWCO 3500 Da) and immersed in 1 L of PBS at 37°C with shaking (50 rpm). At predetermined time points, aliquots were withdrawn and replaced with fresh PBS. Released Sim and GA were quantified by HPLC.

### 4.4 Stability Assay

Colloidal stability of SGNCs in PBS containing 10% FBS at 37°C over 7 days, monitored by DLS. Storage stability of SGNCs as lyophilized powder at 4°C for 180 days, monitored by DLS after reconstitution.

### 4.5 Cell Culture

RAW 264.7 macrophages were cultured in DMEM with 10% FBS and 1% P/S. Rat BMSCs were isolated from 4‑week‑old SD rats, cultured in α‑MEM with 10% FBS and 1% P/S, and used at passages 3–5.

### 4.6 Cellular Uptake and Cytotoxicity

RAW 264.7 macrophages were incubated with FITC‑labeled Sim (10 µg/mL) or SGNCs (50 µg/mL) for 4 h. Lysosomes and nuclei were stained with LysoTracker Red (75 nM) and DAPI (0.1 µg/mL), and visualized by confocal microscopy. Cytotoxicity was assessed by CCK‑8 assay.

### 4.7 ROS Scavenging and Macrophage Polarization

RAW 264.7 cells were pretreated with formulations (GA 40 µg/mL, Sim 10 µg/mL, physical mixture 50 µg/mL, or SGNCs 50 µg/mL) for 12 h, then stimulated with heat‑inactivated *S. aureus* (MOI = 10) or H_2_O_2_ (100 µM) for 12 h. Intracellular ROS was measured using DCFH‑DA by flow cytometry and fluorescence microscopy. For polarization, M1 macrophages were identified as CD86^+^ cells within the F4/80^+^ population by flow cytometry. TNF‑α levels in supernatants were measured by ELISA.

### 4.8 Antibacterial Assay

*S. aureus* (ATCC25923) was cultured to logarithmic phase. The MIC was determined by incubating bacteria with serial twofold dilutions of each formulation in 96‑well plates at 37°C for 24 h. MBC was determined by subculturing aliquots on LB agar plates. Biofilm inhibition was assessed by crystal violet staining.

### 4.9 In Vitro Osteogenic Capacity

BMSCs were exposed to heat‑inactivated *S. aureus* (MOI = 10) and treatments (GA 40 µg/mL, Sim 10 µg/mL, physical mixture 50 µg/mL, or SGNCs 50 µg/mL) for 14 days. ALP and ARS staining were performed using BCIP/NBT and 1% ARS (pH 4.2), respectively. Stained products were quantified by dissolving in 10% cetylpyridinium chloride and measuring absorbance at 405 nm (ALP) or 562 nm (ARS).

### 4.10 qRT‑PCR

Total RNA was extracted using TRIzol. After reverse transcription, qPCR was performed using SYBR Green Master Mix on a QuantStudio 6 Flex system. The expression of Tnf‑α, Tgfb1, Runx2, and Ocn was normalized to Gapdh. Primer sequences are listed in Table S1.

### 4.11 Animal Model

All animal experiments were approved by the Animal Ethics Committee of The First Hospital of Jilin University (Approval No.0392). Male Sprague‐Dawley rats (4 or 8 weeks old) were obtained from Jiangsu Yadong Laboratory Animal Research Institute and housed under specific pathogen‐free conditions with free access to food and water.

A rat tibial osteomyelitis model was established in 8‑week‑old male SD rats. After anesthesia, a 1 mm bone defect was drilled and 30 µL of *S. aureus* (1 × 10^7^ CFU/mL) was injected. Rats were divided into six groups (n = 9 each): control, model, Sim, GA, Sim+GA mixture, and SGNCs. Treatments were administered locally immediately after inoculation. All rats received buprenorphine (0.05 mg/kg) for three days post‑surgery and were euthanized at 4 weeks.

### 4.12 Micro‑CT and In Vivo Antibacterial Effect

Tibial samples were scanned using a SkyScan 1176 µCT system (70 kV, 500 µA, 18 µm voxel size). BV/TV, BMD, Tb.Th, and Tb.Sp were calculated using CTAn. For bacterial load, bone homogenates were plated on LB agar and CFUs counted after 24 h.

### 4.13 Histological and Immunostaining Analysis

Bone tissues were fixed, decalcified in 10% EDTA, and embedded in paraffin. Sections (5 µm) were stained with H&E, Masson's trichrome, or subjected to IHC with anti‑iNOS and anti‑CD206 antibodies. For IF, sections were incubated with anti‑RUNX2 and anti‑OCN antibodies, followed by Alexa Fluor 488/594‑conjugated secondary antibodies. Nuclei were stained with DAPI.

### 4.14 RNA Sequencing

Total RNA from bone marrow samples of all six groups was extracted. Libraries were prepared using the NEBNext Ultra II RNA Library Prep Kit and sequenced on an Illumina NovaSeq 6000 platform (150 bp paired‑end). Clean reads were aligned to the rat genome (Rnor_6.0) using HISAT2. DEGs were identified using DESeq2 (|log_2_FC| > 1, padj < 0.05). KEGG enrichment and GSEA were performed using clusterProfiler and GSEA software, respectively.

### 4.15 Immunofluorescence Validation of Signaling Pathways

For NF‑κB pathway validation, BMSCs were treated with SGNCs, BAY 11‑7082 (NF‑κB inhibitor), or SGNCs plus TNF‑α (rescue) before stimulation with heat‑inactivated *S. aureus*. For Wnt/β‑catenin pathway validation, cells were treated with SGNCs alone, SGNCs plus XAV939 (Wnt inhibitor), or transfected with si‑Ctnnb1 (β‑catenin knockdown) before SGNCs treatment and heat‑inactivated *S. aureus* stimulation. Cells were then fixed and stained with antibodies against p65, β‑catenin, RUNX2, or OCN, followed by fluorescence microscopy. Fluorescence intensity was quantified using ImageJ software.

### 4.16 Biosafety Assessment

Body weights were monitored weekly. Serum ALT, AST, BUN, CREA, and LDH were measured using an automated biochemical analyzer. Major organs were harvested for H&E staining.

### 4.17 In Vivo Fluorescence Imaging

8‐week‐old rats were subjected to the same surgical procedure without bacterial inoculation. Cy5‑labeled SGNCs or Cy5‑labeled Sim+GA mixture were administered locally into the defect. Whole‑body fluorescence images were acquired using an IVIS Spectrum imaging system. Fluorescence intensity at the defect site was quantified using Living Image software. After the final imaging, rats were euthanized, and major organsand the tibia were harvested for ex vivo imaging under the same settings.

### 4.18 Statistical Analysis

Data are presented as mean ± SD. Comparisons between two groups were performed using unpaired two‑tailed Student's t‑test. For multiple comparisons, one‑way ANOVA with Tukey's post hoc test was used. Body weight data collected over multiple time points were analyzed by two‑way repeated‑measures ANOVA with Tukey's post hoc test; the Greenhouse‑Geisser correction was applied when sphericity was violated. Significance levels are indicated as ^*^ *p* < 0.05, ^**^ *p* < 0.01, ^***^ *p* < 0.001.

## Author Contributions

**Yu Han**: conceptualization, funding acquisition, writing – original draft, supervision, methodology. **Yao Zhao**: software, validation. **Chengbo Yang**: validation, formal analysis. **Miao Niu**: investigation, software. **Jun Dai**: investigation, formal analysis. **Quanxin Ning**: validation, methodology, writing – original draft. **Kai Xiao**: methodology, software, data curation. **Jinzheng Liang**: data curation, formal analysis. **Wensheng Zhang**: investigation, data curation. **Liang Wang**: supervision, methodology, writing – review and editing. **Dan Shao**: writing – review and editing, supervision, conceptualization. **Dongsong Li**: writing – review and editing, project administration, funding acquisition.

## Funding

Jilin Scientific and Technological Development Program (YDZJ202401166ZYTS).

## Conflicts of Interest

The authors declare no conflicts of interest.

## Supporting information




**Supporting File**: advs76694‐sup‐0001‐SuppMat.docx.

## Data Availability

The raw RNA‑sequencing data generated in this study have been deposited in the NCBI Sequence Read Archive (SRA) under BioProject accession number [PRJNA1484807], the data will be publicly released upon publication. The data that support the findings of this study are available from the corresponding author upon reasonable request.
