# Supplementary material for: AI‐Assisted Engineering of Glycyrrhizic Acid/Simvastatin Nanocrystals for Multifunctional Treatment of Bacterial Osteomyelitis
Source: Adv Sci (Weinh). 2026 Jul 23;13(57):e76694. doi: 10.1002/advs.76694 (PMC13395250; doi:10.1002/advs.76694)
Supplement: Supplementary file 1 [file ADVS-13-e76694-s001.docx]

Supporting Information

**AI-Assisted Engineering of Glycyrrhizic Acid/Simvastatin Nanocrystals for Multifunctional Treatment of Bacterial Osteomyelitis**

Yu Han, Yao Zhao, Chengbo Yang, Miao Niu, Jun Dai, Quanxin Ning, Kai Xiao, Jinzheng Liang, Wensheng Zhang, Liang Wang*, Dan Shao*, Dongsong Li*

Y. Han, Y. Zhao, C. Yang, M. Niu, J. Dai, D. Li

Department of Orthopaedic Surgery, Orthopaedic Center, The First Hospital of Jilin University, Changchun, Jilin 130021, China

E-mail: [lidongsong@jlu.edu.cn](mailto:lidongsong@jlu.edu.cn)

Q. Ning, K. Xiao, D. Shao

State Key Laboratory of Oral Diseases and National Clinical Research Center for Oral Diseases, West China Hospital of Stomatology, Sichuan University, Chengdu, Sichuan 610041, China.

E-mail: [stanauagate@outlook.com](mailto:stanauagate@outlook.com)

Q. Ning, J. Liang, W. Zhang, L. Wang

Department of Orthopedics, The Third Affiliated Hospital, Southern Medical University, Guangzhou, Guangdong 510630, China.

E-mail: [liang091@aliyun.com](mailto:liang091@aliyun.com)

Detailed Methods

Reagents and materials

Simvastatin (Sim, ≥ 98%), glycyrrhizic acid (GA, ≥ 98%), Crystal Violet (≥ 90%) and Bay 11-7082 (≥ 98%) were purchased from Adamas Reagent Company (Shanghai, China). Ethanol, dimethyl sulfoxide (DMSO) and acetic acid were obtained from Greagent (Shanghai, China). Dialysis bags (molecular weight cut-off: 3500 Da) were purchased from Viskase Companies (Houston, USA). Dulbecco’s modified Eagle’s medium (DMEM), α-minimum essential medium (α-MEM), Opti‑MEM, fetal bovine serum (FBS), penicillin-streptomycin, and trypsin-EDTA were obtained from Gibco (USA). Hydrogen peroxide (H_2_O_2_) was purchased from Cdkelong (Chengdu, China). FITC-Simvastatin and Cy5-Simvastatin were obtained from QIYUE BIOLOGY (Xi’an, China). The Cell Counting Kit-8 (CCK-8) was purchased from APExBIO (Houston, USA). The DCFH-DA reactive oxygen species (ROS) assay kit, LysoTracker Red, and MitoSOX Red were obtained from Beyotime (Jiangsu, China). The BCIP/NBT alkaline phosphatase (ALP) staining kit and Alizarin Red S (ARS) staining kit were purchased from Solarbio (Beijing, China). Enzyme-linked immunosorbent assay (ELISA) kits for tumor necrosis factor-alpha (TNF-α) and interleukin-6 (IL-6) were obtained from BioLegend (San Diego, USA). TRIzol reagent, PrimeScript RT reagent kit and Lipofectamine^TM^ RNAiMAX transfection reagent were purchased from Invitrogen (Carlsbad, CA, USA). SYBR Green Master Mix was obtained from TransGen (China). si‑Ctnnb1 were purchased from Sangon Biotech (Shanghai, China). Primary antibodies against F4/80 (FITC-conjugated), CD86 (PE-conjugated), iNOS, CD206, p65, β-catenin, RUNX2, OCN and XAV939 were purchased from Abcam (UK) or Proteintech (China). Secondary antibodies conjugated with horseradish peroxidase (HRP) or Alexa Fluor dyes (488 and 594) were obtained from Invitrogen (USA). DAPI was purchased from Beyotime (Shanghai, China). Luria-Bertani (LB) broth and LB agar were purchased from Solarbio (Beijing, China). For RNA sequencing, the NEBNext Ultra II RNA Library Prep Kit (New England Biolabs, USA) was used. All other chemicals were of analytical grade and used without further purification.

All animal experiments were approved by the Animal Ethics Committee of The First Hospital of Jilin University (Approval No.0392). Male Sprague-Dawley rats (4 or 8 weeks old) were obtained from Jiangsu Yadong Laboratory Animal Research Institute and housed under specific pathogen-free conditions with free access to food and water.

**Bioinformatics-based target identification and pathway enrichment analysis**

Disease-associated genes for bacterial osteomyelitis were retrieved from GeneCards using "bacterial osteomyelitis" as query terms, followed by deduplication.^[1]^ Kyoto Encyclopedia of Genes and Genomes (KEGG) pathway enrichment and protein–protein interaction (PPI) networks analysis was performed using STRING.^[2, 3]^ The top five targets (IL6, NFKBIA, IL1B, TNF, and NFKB1) were queried in Comparative Toxicogenomics Database (CTD),^[4]^ and results filtered for FDA-approved natural compounds, yielding 16 immunomodulatory candidates. Separately, 9 FDA-approved small molecules with documented pro-osteogenic activity were curated from the literature and DrugBank.^[5]^ Pairwise combination of both libraries generated 144 binary drug combinations for subsequent nanocrystal screening.

**Machine learning-guided prediction of nanocrystal-forming drug pairs**

A binary-labeled dataset of 200 drug pairs was assembled from two sources. Literature-derived samples (n = 83) were collected from published studies reporting experimentally validated excipient-free nanoparticle formation or non-assembly outcomes, with labels assigned according to the original reported results. Experimentally generated samples (n = 117) were prepared in the present work as follows: compounds A and B were co-dissolved in DMSO at a concentration of 5 mM each. 0.05 mL solution was rapidly added to 1 mL deionized water, followed by vortex mixing.

Molecular descriptors for each compound were calculated using RDKit, and the descriptor vectors of each drug pair were concatenated as model input. Multiple classifiers were evaluated under 10-fold cross-validation, including XGBoost (XGB), K-nearest neighbours (KNN), Gaussian Naive Bayes (GNB), support vector machine (SVM), and decision tree (DT). Model performance was assessed by accuracy, F1-score, average precision, area under the ROC curve (AUC-ROC), area under the precision-recall curve (AUC-PR), and calibration curve analysis; recall, precision, and Matthews correlation coefficient (MCC) are reported in the Supplementary Information. XGBoost achieved the highest overall performance and was selected for prospective screening of the 144-candidate combination library. An independent external validation set of 89 literature-reported nanoassembly-forming API pairs, not included in model training, was used to evaluate generalizability. Model interpretability was assessed using SHAP analysis.^[6]^

**Preparation and characterization**

Simvastatin (Sim) and glycyrrhizic acid (GA) were dissolved in ethanol and deionized water, respectively, at a molar ratio of 1:2 (Sim:GA). The two solutions were rapidly mixed using flash nanoprecipitation to induce co-assembly. The resulting suspension was dialyzed (MWCO: 3500 Da) against deionized water for 24 h to remove organic solvents, followed by lyophilization to obtain SGNCs powder. Based on the negligible mass loss during preparation and the complete incorporation of both components confirmed by subsequent characterization, the mass ratio of Sim to GA in the final SGNCs was calculated to be approximately 1:4, consistent with the initial molar ratio.

Morphology was examined by transmission electron microscopy (TEM; JEM-2100F, JEOL, Japan) at 200 kV. High-resolution TEM was used to visualize lattice fringes. Hydrodynamic diameter and zeta potential were measured by dynamic light scattering (DLS; Zetasizer Nano ZS, Malvern, UK). Crystalline structures were characterized by X-ray diffraction (XRD; D8 ADVANCE, Bruker, Germany) over 5–90° (2θ) at 2°/min. Fourier-transform infrared (FT-IR) spectra were recorded on a spectrometer (Nicolet 6700, Thermo Fisher, USA) over 400-4000 cm^-1^.

**Quantum chemical calculations and Molecular dynamics simulations**

Single-point calculations for compounds Sim and GA were performed using ORCA 6.0 at the r2SCAN-3c level of theory to obtain atomic charge distributions and wavefunction information.^[7, 8]^ Electrostatic potential (ESP) maps were generated from the resulting wavefunctions to visualize the charge complementarity between the two molecules. Low-energy binding conformations of the Sim+GA complex were systematically explored and optimized using MOPAC,^[9]^ with conformer sampling and clustering performed using Molclus in conjunction with Multiwfn. Intermolecular interactions within the optimized binding conformations were visualized and analyzed using the independent gradient model based on Hirshfeld partition (IGMH) method as implemented in Multiwfn,^[10]^ with VMD employed for three-dimensional rendering.^[11]^

Molecular topology files for Sim and GA were constructed using Sobtop, with atomic partial charges derived directly from the preceding ORCA single-point calculations. The generalized AMBER force field (GAFF) was applied to both compounds.^[12]^ The simulation system was constructed by randomly placing 25 molecules of Sim and 50 molecules of GA in a cubic box of 10 × 10 × 10 nm, followed by solvation with TIP3P explicit water molecules. All MD simulations were performed using GROMACS 2025.2.^[13]^ The system was first subjected to energy minimization using the steepest descent algorithm, followed by sequential equilibration in the NVT and NPT ensembles for 100 ps each, with temperature maintained at 300 K using the V-rescale thermostat and pressure controlled at 1 bar using the Parrinello–Rahman barostat. Production simulations were carried out for 600 ns with an integration time step of 2 fs. Trajectory analyses, including cluster structure characterization, radius of gyration, solvent-accessible surface area (SASA), pairwise interaction energy decomposition and free energy surfaces (FES) were performed using built-in GROMACS tools. Molecular hydrophobicity distribution maps were generated by calculating atomic contributions to LogP using rdkit and visualized in conjunction with the cluster structures to assess the role of hydrophobic interactions in the co-assembly process.

***In vitro* drug release behavior**

For *in vitro* drug release studies, 1 mg SGNCs were dispersed in 10 mL of phosphate-buffered saline (PBS; pH 7.4) and placed into dialysis bags (MWCO: 3500 Da). The bags were immersed in 1 L of PBS and incubated at 37 °C with continuous shaking at 50 rpm. At predetermined time points (0, 0.5, 1, 2, 4, 6, 8, 12, 24, 48 h), aliquots of 0.1 mL were withdrawn and replaced with fresh PBS. The concentrations of Sim and GA were quantified by high-performance liquid chromatography (HPLC; 1260 Infinity II, Agilent, USA). All experiments were performed in triplicate.

**Stability assay**

SGNCs were dispersed in PBS containing 10% FBS at a final concentration of 1 mg/mL and incubated at 37 °C with gentle shaking (100 rpm). At predetermined time points (1, 2, 3, 4, 5, 6, 7 days), aliquots were withdrawn and the hydrodynamic diameter and polydispersity index were measured by DLS. SGNCs were stored as lyophilized powder at 4 °C for up to 180 days. The powder was reconstituted in PBS and analyzed by DLS for particle size and PDI. All measurements were performed in triplicate.

**Cell cultures**

RAW 264.7 macrophages were cultured in Dulbecco's modified Eagle's medium supplemented with 10% fetal bovine serum and 1% penicillin-streptomycin at 37 °C in a humidified atmosphere containing 5% CO_2_. Bone marrow-derived mesenchymal stem cells (BMSCs) were isolated from the femurs and tibias of 4-week-old male Sprague-Dawley rats. Briefly, bone marrow cavities were flushed with alpha minimum essential medium, and the collected cells were cultured in α-MEM containing 10% FBS and 1% P/S. Non-adherent cells were removed after 24 h, and adherent BMSCs were expanded and used at passages 3-5 for all experiments. Cells were maintained at 37 °C in a 5% CO_2_ incubator with medium changed every 2 days. The osteogenic differentiation medium was added with dexamethasone at 10 nM, ascorbic acid at 50 μg/mL and β-glycerophosphate at 10 mM.^[14]^

**Cellular uptake**

RAW 264.7 macrophages were seeded on glass-bottom confocal dishes at a density of 1 × 10^5^ cells per dish and cultured overnight. FITC-labeled Sim or FITC-labeled SGNCs were prepared at a concentration of 10 μg/mL or 50 μg/mL and incubated with the cells for 4 h at 37 °C. For lysosomal colocalization, cells were stained with LysoTracker Red (75 nM, 30 min) and nuclei were counterstained with DAPI (0.1 μg/mL, 5 min). After washing with PBS, the cells were immediately visualized under a confocal laser scanning microscope.

**Cytotoxicity assessment**

The cytotoxicity of SGNCs was evaluated using the Cell Counting Kit-8 (CCK-8) assay. BMSCs were seeded in 96-well plates at a density of 5 × 10^3^ cells per well and cultured overnight at 37 °C in a 5% CO_2_ incubator. The cells were then treated with various concentrations of free GA, free Sim, Sim+GA physical mixture, or SGNCs for 24 h or 48 h. After treatment, the culture medium was replaced with 100 μL of fresh medium containing 10% CCK-8 reagent, and the plates were incubated for an additional 2 h at 37 °C. The absorbance was measured at 450 nm using a microplate reader. Cell viability was calculated relative to untreated control cells, and all experiments were performed in triplicate.

**ROS scavenging**

Intracellular reactive oxygen species (ROS) levels were evaluated using the DCFH-DA fluorescent probe. RAW 264.7 macrophages were seeded in 6-well plates at a density of 2 × 10^5^ cells per well and cultured overnight at 37 °C in a 5% CO_2_ incubator. Cells were pretreated with various formulations (free GA 40 μg/mL, free Sim 10 μg/mL, Sim+GA physical mixture 50 μg/mL, or SGNCs 50 μg/mL) for 12 h. To establish an oxidative stress model, cells were then exposed to heat-inactivated *S. aureus* (MOI = 10) or H_2_O_2_ (100 μM) for 12 h. Subsequently, cells were washed twice with PBS and incubated with DCFH-DA (10 μM) in serum-free medium at 37 °C for 20 min in the dark. After three washes with PBS to remove extracellular probe, ROS levels were immediately analyzed using a flow cytometer with excitation at 488 nm and emission at 525 nm. For fluorescence imaging, cells were seeded on glass-bottom confocal dishes and stained following the same protocol, then visualized under a fluorescence microscope. Fluorescence intensity was quantified using ImageJ software, and all experiments were performed in triplicate.

**Macrophage polarization**

RAW 264.7 macrophages were seeded in 6-well plates at a density of 5 × 10^5^ cells per well and cultured overnight at 37 °C in a 5% CO_2_ incubator. Cells were pretreated with various formulations (free GA 40 μg/mL, free Sim 10 μg/mL, Sim+GA physical mixture 50 μg/mL, or SGNCs 50 μg/mL) for 12 h. To induce M1 polarization, cells were then stimulated with heat-inactivated *S. aureus* (MOI = 10) for 12 h. After treatment, cells were harvested and washed twice with PBS. For flow cytometric analysis, cells were incubated with FITC-conjugated anti-F4/80 antibody (1:200) and PE-conjugated anti-CD86 antibody (1:200) for 30 min at 4 °C in the dark. After washing, cells were resuspended in PBS and analyzed using a flow cytometer. The proportion of M1 macrophages was determined as the percentage of CD86^+^ cells within the F4/80^+^ population. All experiments were performed in triplicate.

**Antibacterial assay**

The antibacterial activity of SGNCs was evaluated against *Staphylococcus aureus* (ATCC25923). Briefly, *S. aureus* was cultured overnight in Luria-Bertani (LB) broth at 37 °C with shaking at 220 rpm to reach the logarithmic growth phase. The bacterial suspension was then diluted with fresh LB broth to a concentration of approximately 1 × 10^7^ colony-forming units (CFU)/mL. Serial two-fold dilutions of free GA, free Sim, Sim+GA physical mixture, and SGNCs were prepared in 96-well microplates, ranging from 19.53 to 625 μg/mL. Each well received 100 μL of bacterial suspension, and the plates were incubated at 37 °C for 24 h. The minimum inhibitory concentration (MIC) was defined as the lowest concentration of each formulation that completely inhibited visible bacterial growth. To determine the minimum bactericidal concentration (MBC), 10 μL aliquots from wells were spread onto LB agar plates and incubated at 37 °C for 24 h. The MBC was defined as the lowest concentration that killed ≥99.9% of the initial bacterial inoculum. All experiments were performed in triplicate.

**Biofilm inhibition assay**

*S. aureus* was cultured overnight in LB broth at 37 ℃ with shaking (220 rpm). The bacterial suspension was diluted to 1 × 10^6^ CFU/mL in fresh LB broth, and 1 mL was added to each well of a 24‑well plate. After incubation at 37 ℃ for 72 h (with medium refreshed every 24 h), the supernatant was removed, and the wells were gently washed twice with sterile PBS to remove planktonic bacteria. Then, 1 mL of each formulation (SGNCs, Sim+GA mixture, Sim, GA, or PBS as control) at a concentration equivalent to 156.25 μg/mL of SGNCs was added and incubated for another 24 h at 37 ℃. After treatment, the medium was discarded, and the wells were washed three times with PBS. Biofilms were fixed with methanol for 15 min, air‑dried, and stained with 0.1% (w/v) crystal violet for 15 min at room temperature. Excess stain was removed by gentle washing with PBS, and the bound dye was solubilized with 33% (v/v) glacial acetic acid. The absorbance was measured at 595 nm using a microplate reader. Biofilm biomass was expressed as percentage relative to the untreated control (set as 100%). All experiments were performed in triplicate.

**Osteogenic capacity *in vitro***

To evaluate the osteogenic capacity under infectious conditions, BMSCs were seeded in 24-well plates at a density of 2 × 10^4^ cells per well and exposed to heat-inactivated *S. aureus* (MOI = 10) with or without various treatments (free GA 40 μg/mL, free Sim 10 μg/mL, Sim+GA physical mixture 50 μg/mL, or SGNCs 50 μg/mL) for 14 days. Untreated cells without bacterial exposure served as control. The culture medium was changed every 2-3 days.

For alkaline phosphatase (ALP) staining and quantification, cells were fixed with 4% paraformaldehyde for 15 min at day 14, then stained using a BCIP/NBT ALP staining kit according to the manufacturer's instructions. For quantitative analysis, the stained dye was dissolved in 10% cetylpyridinium chloride, and absorbance was measured at 405 nm using a microplate reader. For Alizarin Red S (ARS) staining, cells were fixed with 4% paraformaldehyde for 15 min, stained with 1% ARS solution (pH 4.2) for 30 min at room temperature, and washed with deionized water. For quantitative analysis, the stained nodules were dissolved in 10% cetylpyridinium chloride, and absorbance was measured at 562 nm.

**qRT-PCR assessment**

Total RNA was extracted from BMSCs after 14 days of treatment using TRIzol reagent according to the manufacturer's protocol. RNA concentration and purity were determined using a NanoDrop spectrophotometer. Reverse transcription was performed using a PrimeScript RT reagent kit with 1 μg of total RNA following the manufacturer's instructions. Quantitative real-time PCR was conducted using SYBR Green Master Mix on a QuantStudio^TM^ 6 Flex Real-Time PCR System (Thermo Fisher Scientific, USA). The thermal cycling conditions were as follows: 95 °C for 2 min, followed by 40 cycles of 95 °C for 15 s and 60 °C for 30 s. The expression levels of Tnf-α, Tgfb1, Runx2, and Ocn were quantified and normalized to the housekeeping gene Gapdh. The primer sequences used in this study are listed in Table S1. Relative gene expression was calculated using the 2^-ΔΔCt^ method. All reactions were performed in triplicate.

**Animal models**

Rats were anesthetized by intraperitoneal injection of pentobarbital sodium (50 mg/kg). The right hind limb was shaved and sterilized with 75% ethanol and iodine. A 1 cm longitudinal incision was made over the proximal tibia, and the periosteum was carefully dissected to expose the tibial plateau. A bone defect (1 mm in diameter) was created using a sterile dental drill under continuous saline irrigation to minimize thermal necrosis. A 30 μL suspension of *S. aureus* containing 1 × 10^7^ CFU/mL was injected into the bone defect. For the control group, 10 μL of sterile PBS was injected instead of bacterial suspension. The wound was then sutured layer by layer.^[15]^ Rats were randomly divided into six groups (n = 9 per group): control, model, Sim, GA, Sim+GA physical mixture, and SGNCs. All treatments were administered locally via injection into the bone defect immediately after bacterial inoculation. All rats received intramuscular injections of buprenorphine (0.05 mg/kg) for three consecutive days post-surgery to relieve pain. Animals were euthanized at 4 weeks post-surgery for subsequent analyses. The experimental procedure is schematically illustrated in Figure S16.

**Microcomputed imaging (μCT)**

Tibial samples were harvested at 4 weeks post-surgery, fixed in 4% paraformaldehyde for 48 h, and stored in 70% ethanol prior to scanning. μCT imaging was performed using a SkyScan 1176 micro-CT system (Bruker, Germany) with the following parameters: X-ray source voltage of 70 kV, current of 500 μA, aluminum filter of 0.5 mm, and isotropic voxel size of 18 μm. The samples were scanned over a 180° rotation with a rotation step of 0.5°.

Three-dimensional reconstruction and quantitative analysis were performed using NRecon and CTAn software. A cylindrical region of interest (ROI) with a diameter of 2 mm and height of 1.5 mm was selected at the bone defect site, encompassing the entire defect area. The following trabecular bone parameters were calculated: bone volume fraction (BV/TV, %), bone mineral density (BMD, g/cm^3^), trabecular thickness (Tb.Th, mm), and trabecular separation (Tb.Sp, mm). All analyses were performed following established guidelines for bone microarchitecture evaluation.

**Antibacterial effect *in vivo***

To evaluate the antibacterial efficacy of SGNCs *in vivo*, the bone defect region was harvested at 4 weeks post-surgery under sterile conditions. The tibial bone samples were weighed and homogenized in sterile PBS at a ratio of 0.1 g/mL using a tissue homogenizer. The homogenates were diluted 10-fold with sterile PBS, and 50 μL aliquots from each dilution were spread onto LB agar plates. The plates were incubated at 37 °C for 24 h, and the number of CFU was counted. All experiments were performed in triplicate for each sample.

**Histological and immunostaining analysis**

Tibial samples harvested at 4 weeks post-surgery were fixed in 4% paraformaldehyde for 48 h, decalcified in 10% EDTA (pH 7.4) for 4 weeks at room temperature with gentle shaking, dehydrated through a graded ethanol series, cleared in xylene, and embedded in paraffin. Serial sections (5 μm thick) were cut using a microtome (Leica RM2255, Germany).

For hematoxylin and eosin (H&E) staining, sections were deparaffinized, rehydrated, stained with hematoxylin for 5 min and eosin for 2 min, then dehydrated and mounted. For Masson’s trichrome staining, sections were stained following the manufacturer’s protocol to visualize collagen deposition. Images were captured using an optical microscope (Olympus BX53, Japan).

For immunohistochemical staining, sections were deparaffinized, rehydrated, and subjected to antigen retrieval by heating in citrate buffer (pH 6.0) for 10 min. Endogenous peroxidase activity was blocked with 3% H_2_O_2_ for 10 min, followed by blocking with 5% BSA for 1 h at room temperature. Sections were incubated with primary antibodies against iNOS (1:200) and CD206 (1:200) overnight at 4 °C. After washing, sections were incubated with horseradish peroxidase-conjugated secondary antibodies for 1 h at room temperature, followed by DAB substrate for color development. Sections were counterstained with hematoxylin, dehydrated, and mounted. Images were captured using an optical microscope.

For immunofluorescence staining, sections were deparaffinized, rehydrated, and subjected to antigen retrieval as described above. After blocking with 5% BSA for 1 h, sections were incubated with primary antibodies against RUNX2 (1:200) and OCN (1:200) overnight at 4 °C. After washing, sections were incubated with Alexa Fluor 488-conjugated secondary antibody (1:500) for RUNX2 and Alexa Fluor 594-conjugated secondary antibody (1:500) for OCN for 1 h at room temperature in the dark. Nuclei were counterstained with DAPI (0.1 μg/mL, 5 min). Fluorescence images were captured using a fluorescence microscope.

**RNA sequencing and analysis**

Total RNA was extracted from bone marrow samples collected from each experimental group (control, model, Sim, GA, Sim+GA physical mixture, and SGNCs) using TRIzol reagent according to the manufacturer’s protocol. RNA concentration and purity were assessed using a NanoDrop 2000 spectrophotometer, and RNA integrity was evaluated using an Agilent 2100 Bioanalyzer. Samples with RNA integrity number (RIN) ≥ 8.0 were used for subsequent library preparation.

RNA sequencing libraries were constructed using the NEBNext Ultra II RNA Library Prep Kit following the manufacturer’s instructions. Sequencing was performed on an Illumina NovaSeq 6000 platform (Illumina, USA) with paired-end reads of 150 bp. Raw sequencing reads were processed to remove adapter sequences and low-quality bases using Trimmomatic software. Clean reads were aligned to the rat reference genome (Rnor_6.0) using HISAT2 software. Gene expression levels were quantified as fragments per kilobase of transcript per million mapped reads (FPKM) using featureCounts software.

Differentially expressed genes (DEGs) between groups were identified using DESeq2 software with thresholds of |log_2_ fold change| > 1 and adjusted p-value < 0.05. Kyoto Encyclopedia of Genes and Genomes (KEGG) pathway enrichment analyses were performed using clusterProfiler package in R. Gene set enrichment analysis (GSEA) was conducted using GSEA software (Broad Institute) with the Molecular Signatures Database (MSigDB), and gene sets with false discovery rate (FDR) < 0.25 and p-value < 0.05 were considered significantly enriched.

**siRNA transfection**

BMSCs were seeded in 6‑well plates at a density of 2 × 10^5^ cells per well and cultured in α‑MEM with 10% FBS until reaching 50-70% confluence. For transfection, 2.5 μL of Lipofectamine RNAiMAX was diluted in 125 μL Opti‑MEM, and 5 μL of 20 μM siRNA stock (final concentration 50 nM) was diluted in 125 μL Opti‑MEM. The two solutions were mixed and incubated at room temperature for 15 min to form lipid‑siRNA complexes. The mixture was then added dropwise to the cells, and the plates were gently swirled. After 12 h of incubation at 37 °C in 5% CO_2_, the medium was replaced with fresh α‑MEM containing 10% FBS.

**Immunofluorescence staining for signaling pathway validation**

BMSCs were seeded in 24‑well plates at a density of 5 × 10^4^ cells/well. For NF‑κB pathway validation, BMSCs were pretreated with SGNCs (50 μg/mL), BAY 11‑7082 (10 μM, NF‑κB inhibitor), or SGNCs plus TNF‑α (20 ng/mL, rescue) for 30 min, then stimulated with heat‑inactivated *S. aureus* (MOI = 10) for 24 h. For Wnt/β‑catenin pathway validation, BMSCs were pretreated with SGNCs (50 μg/mL), SGNCs plus XAV939 (5 μM, Wnt inhibitor), or first transfected with si‑Ctnnb1 (β‑catenin knockdown) or si‑NC (negative control) as described above and then treated with SGNCs (50 μg/mL). All groups were subsequently stimulated with heat‑inactivated *S. aureus* (MOI = 10) for 24 h.

After the indicated treatments, cells were washed twice with PBS, fixed with 4% paraformaldehyde for 15 min at room temperature, and permeabilized with 0.1% Triton X‑100 in PBS for 10 min. Nonspecific binding was blocked with 5% BSA in PBS for 1 h. Subsequently, cells were incubated overnight at 4 °C with primary antibodies: anti‑p65 (1:200), anti‑β‑catenin (1:200), anti‑RUNX2 (1:200), or anti‑OCN (1:200). After washing three times with PBS, cells were incubated with appropriate Alexa Fluor 488-conjugated secondary antibody (1:500) or Alexa Fluor 594-conjugated secondary antibody (1:500) for 1 h at room temperature in the dark. Nuclei were counterstained with DAPI (0.1 μg/mL) for 5 min. Coverslips were mounted on slides with anti‑fade mounting medium. Fluorescence images were acquired using a confocal laser scanning microscope. Fluorescence intensity was quantified using ImageJ software.

**Biosafety assessment**

To evaluate the *in vivo* biocompatibility of SGNCs, body weight changes of all rats were monitored weekly throughout the 4-week treatment period. For body weight data collected repeatedly over time, two‑way repeated‑measures ANOVA with treatment and time as factors was performed, followed by Tukey’s post hoc test for multiple comparisons. The Greenhouse‑Geisser correction was applied when the sphericity assumption was violated. At the time of euthanasia, blood samples were collected via cardiac puncture and centrifuged at 3000 rpm for 10 min to obtain serum. Serum biochemical parameters, including alanine aminotransferase (ALT), aspartate aminotransferase (AST), blood urea nitrogen (BUN), creatinine (CREA), and lactatedehydrogenase (LDH) were measured using an automated biochemical analyzer following the manufacturer’s instructions.

For histopathological examination, major organs (heart, liver, spleen, lung, and kidney) were harvested at 4 weeks post-surgery, fixed in 4% paraformaldehyde for 48 h, dehydrated through a graded ethanol series, cleared in xylene, and embedded in paraffin. Sections (5 μm thick) were cut and stained with H&E following standard protocols. Images were captured using an optical microscope.

***In vivo* fluorescence imaging**

SD rats (8 weeks old, 220-250 g, n = 3 per group) were anesthetized with pentobarbital sodium (50 mg/kg i.p.). The right hind limb was shaved, and a 1 mm bone defect was drilled in the proximal tibia. The defect was sealed with bone wax and sutured. Cy5‑labeled SGNCs or Cy5‑labeled Sim+GA mixture was administered locally. Whole‑body fluorescence images were acquired with an IVIS Spectrum (PerkinElmer, USA) at 12, 24, 48, 72 h and 7 days post‑injection (excitation: 488 nm, emission: 520 nm, exposure time: 0.5 s, binning: 8). Radiant efficiency at the tibial defect was quantified using Living Image 4.0 software. At 7 days, rats were euthanized, the heart, liver, spleen, lung, kidney, and the tibia were harvested. *Ex vivo* images were acquired under the same imaging parameters. All procedures were performed under isoflurane anesthesia during imaging.

**References**

[1] G. Stelzer, N. Rosen, I. Plaschkes, S. Zimmerman, M. Twik, S. Fishilevich, T. I. Stein, R. Nudel, I. Lieder, Y. Mazor, S. Kaplan, D. Dahary, D. Warshawsky, Y. Guan-Golan, A. Kohn, N. Rappaport, M. Safran, D. Lancet, “The GeneCards Suite: From Gene Data Mining to Disease Genome Sequence Analyses,” *Curr. Protoc. Bioinform.* 54 (2016): 1.30.1.

<https://doi.org/10.1002/cpbi.5>

[2] D. Szklarczyk, R. Kirsch, M. Koutrouli, K. Nastou, F. Mehryary, R. Hachilif, A. L. Gable, T. Fang, Nadezhda T. Doncheva, S. Pyysalo, P. Bork, Lars J. Jensen, C. von Mering, “The STRING Database in 2023: Protein-Protein Association Networks and Functional Enrichment Analyses for Any Sequenced Genome of Interest,” *Nucleic Acids Res.* 51 (2023): 638.

<https://doi.org/10.1093/nar/gkac1000>

[3] M. Kanehisa, M. Furumichi, M. Tanabe, Y. Sato, K. Morishima, “KEGG: New Perspectives on Genomes, Pathways, Diseases and Drugs,” *Nucleic Acids Res.* 45 (2023): 353.

[4] A. P. Davis, T. C. Wieger, R. J. Johnson, D. Sciaky, J. Wiegers, C. J. Mattingly, “Comparative Toxicogenomics Database (CTD): Update 2023,” *Nucleic Acids Res.* 51 (2023): 1257.

<https://doi.org/10.1093/nar/gkac833>

[5] C. Knox, M. Wilson, C. M. Klinger, M. Franklin, E. Oler, A. Wilson, A. Pon, J. Cox, N. E. Chin, S. A. Strawbridge, M. Garcia-Patino, R. Kruger, A. Sivakumaran, S. Sanford, R. Doshi, N. Khetarpal, O. Fatokun, D. Doucet, A. Zubkowski, D. Y. Rayat, H. Jackson, K. Harford, A. Anjum, M. Zakir, F. Wang, S. Tian, B. Lee, J. Liigand, H. Peters, R. Q. Wang, T. Nguyen, D. So, M. Sharp, R. da Silva, C. Gabriel, J. Scantlebury, M. Jasinski, D. Ackerman, T. Jewison, T. Sajed, V. Gautam, D. S. Wishart, “DrugBank 6.0: The DrugBank Knowledgebase for 2024,” *Nucleic Acids Res.* 52, (2024): 1265.

<https://doi.org/10.1093/nar/gkad976>

[6] D. Reker, Y. Rybakova, A. R. Kirtane, R. Cao, J. Yang, N. Navamajiti, A. Gardner, R. M. Zhang, T. Esfandiary, J. L’Heureux, T. Erlach, E. M. Smekalova, D. Leboeuf, K. Hess, A. Lopes, J. Rogner, J. Collins, S. M. Tamang, K. Ishida, P. Chamberlain, D. Yun, A. Lytton-Jean, C. K. Soule, J. H. Cheah, A. M. Hayward, R. Langer, G. Traverso, “Computationally Guided High-Throughput Design of Self-assembling Drug Nanoparticles,” *Nat. Nanotechnol.* 16 (2021): 725.

<https://doi.org/10.1038/s41565-021-00870-y>

[7] F. Neese, F. Wennmohs, U. Becker, C. Riplinger, “The ORCA Quantum Chemistry Program Package,” *J. Chem. Phys.* 152 (2020): 224108.

<https://doi.org/10.1063/5.0004608>

[8] S. Grimme, A. Hansen, S. Ehlert, J. Mewes, “r^2^SCAN-3c: A “Swiss Army Knife” Composite Electronic-Structure Method,” *J. Chem. Phys.* 154 (2021): 064103.

<https://doi.org/10.1063/5.0040021>

[9] J. J. P. Stewart, “MOPAC: A Semiempirical Molecular Orbital Program,” *J. Comput.-Aided Mol. Des.* 4 (1990): 1.

<https://doi.org/10.1007/BF00128336>

[10] T. Lu, Q. Chen, “Independent Gradient Model Based on Hirshfeld Partition: A New Method for Visual Study of Interactions in Chemical Systems,” *J. Comput. Chem.* 43 (2022): 539.

<https://doi.org/10.1002/jcc.26812>

[11] W. Humphrey, A. Dalke, K. Schulten, “VMD: Visual Molecular Dynamics,” *J. Mol. Graph.* 14 (1996): 33.

<https://doi.org/10.1016/0263-7855(96)00018-5>

[12] J. Wang, R. M. Wolf, J. W. Caldwell, P. A. Kollman, D. A. Case, “Development and Testing of a General Amber Force Field,” *J. Comput. Chem.* 25 (2022): 1157.

<https://doi.org/10.1002/jcc.20035>

[13] M. J. Abraham, T. Murtola, R. Schulz, S. Páll, J. C. Smith, B. Hess, E. Lindahl, “GROMACS: High Performance Molecular Simulations through Multi-Level Parallelism from Laptops to Supercomputers,” *SoftwareX* 1 (2015): 19.

<https://doi.org/10.1016/j.softx.2015.06.001>

[14] J. Chen, G. Xian, Z. Xiao, F. Ge, S. Yuan, B. Li, X. Liang, Z. Cai, N. Zhang, L. Zhang, Z. A. Li, L. Deng, C. Zeng, D. Xie, “Biomineralization-Inspired Scaffolds Using Citrate-Based Polymers to Stabilize Amorphous Calcium Phosphate Promote Osteogenesis and Angiogenesis for Bone Defect Repair,” *Bioact. Mater.* 56 (2026): 260.

<https://doi.org/10.1016/j.bioactmat.2025.10.016>

[15] Y. Zhang, Y. Cheng, Z. Zhao, S. Jiang, Y. Zhang, J. Li, S. Huang, W. Wang, Y. Xue, A. Li, Z. Tao, Z. Wu, X. Zhang, “Enhanced Chemoradiotherapy for MRSA-Infected Osteomyelitis Using Immunomodulatory Polymer-Reinforced Nanotherapeutics,” *Adv. Mater.* 36 (2024): 2304991.

<https://doi.org/10.1002/adma.202304991>

**Figure S1.** Chemical structures of immunomodulatory candidate molecules.

Chemical structures of 16 FDA‑approved natural products identified from the CTD database targeting IL6, NFKBIA, IL1B, TNF, and NFKB1.

**Figure S2.** Chemical structures of pro‑osteogenic candidate molecules.

Chemical structures of 9 FDA‑approved small molecules with documented pro‑osteogenic activity curated from DrugBank and literature.

**Figure S3.** Performance of different machine learning models in 10‑fold cross‑validation.

**a-c,** Recall (a), precision (b), and Matthews correlation coefficient (c) of XGBoost, KNN, GNB, SVM, and DT models. Data are shown as mean ± SD (n = 10). **p < 0.01, ***p < 0.001.

**Figure S4.** ROC curves of different machine learning models.

**a-c,** Mean ROC curves (shaded areas: ± SD) of GNB (a), SVM (b), and DT (c) from 10‑fold cross‑validation.

**Figure S5.** Precision‑recall curves of different machine learning models.

**a-c,** Mean PR curves (shaded areas: ± SD) of GNB (a), SVM (b), and DT (c) from 10‑fold cross‑validation.

**Figure S6.** Calibration curves of different machine learning models.

**a-c,** Calibration curves of GNB (a), SVM (b), and DT (c) from 10‑fold cross‑validation. x‑axis: mean predicted probability; y‑axis: fraction of positives.

**Figure S7.** SHAP analysis for model interpretability.

**a,** SHAP summary plot showing the distribution and direction of feature contributions across all samples. **b,** Ranking of molecular descriptors according to their mean absolute SHAP values, indicating their overall contribution to model predictions.

**Figure S8.** External validation of the XGBoost model on literature‑reported nanoassembly systems.

Prediction scores for an external validation set comprising 89 literature-reported nanoassembly systems not included in model training. Among them, 65 combinations were correctly classified as positive, supporting the model's generalizability to previously unseen drug-excipient pairs.

**Figure S9.** Stability characterization of SGNCs.

**a,** Colloidal stability of SGNCs in PBS containing 10% FBS at 37 °C over 7 days, monitored by DLS. **b,** Storage stability of SGNCs as lyophilized powder at 4 °C for 180 days. After reconstitution, monitored by DLS. Data are shown as mean ± SD (n = 3).

**Figure S10.** Analysis of intermolecular interactions during MD simulations.

**a,** Number of hydrogen bonds between Sim-Sim, Sim-GA, and GA-GA over 600 ns. **b,** Time‑dependent changes in contacting atom pairs (distance < 3.5 Å).

**Figure S11.** Hydrophobicity mapping of individual molecules.

**a-b,** Hydrophobic surface maps of GA (a) and Sim (b) colored according to calculated LogP (blue: hydrophilic, red: hydrophobic).

**Figure S12.** Cellular uptake of free Sim and SGNCs.

Confocal images of RAW 264.7 macrophages showing uptake of free Sim (left) and SGNCs (right). Lysosomes in red, nuclei in blue, FITC‑labeled drugs in green.

**Figure S13.** Flow cytometry analysis of ROS levels.

Quantitative analysis of ROS levels in RAW 264.7 macrophages after different treatments. Data are shown as mean ± SD (n = 3). ***p < 0.001.

**Figure S14.** Antibacterial activity assessed by colony formation.

Representative images of bacterial colonies on LB agar plates for MBC assays after 24 h. Serial twofold dilutions of each formulation were tested.

**Figure S15.** Biofilm inhibition assay by crystal violet staining.

**a,** Representative images of crystal violet‑stained biofilms formed by *S. aureus*.

**b,** Quantitative analysis of biofilm biomass expressed as percentage relative to the *S. aureus* alone group (set as 100%). Data are shown as mean ± SD (n = 3). ***p < 0.001.

**Figure S16.** Schematic diagram of the animal experimental procedure.

**a-b,** Establishment and administration of the rat tibial osteomyelitis model (a) and experimental timeline (b).

**Figure S17.** Quantitative analysis of trabecular separation (Tb.Sp).

Quantitative analysis of Tb.Sp from micro‑CT data shown in Figure 5a. Data are shown as mean ± SD (n = 3). ***p < 0.001.

**Figure S18.** Quantitative analysis of collagen deposition.

Quantitative analysis of collagen area from Masson’s trichrome staining shown in Figure 6b. Data are shown as mean ± SD (n = 3). *p < 0.05, ***p < 0.001.

**Figure S19.** Quantitative analysis of iNOS and CD206 expression.

**a-b,** Quantitative analysis of iNOS‑positive (a) and CD206‑positive (b) cells from immunohistochemical staining shown in Figure 6c. Data are shown as mean ± SD (n = 3). ***p < 0.001.

**Figure S20.** Immunofluorescence staining of bone sections.

Representative immunofluorescence images of bone sections showing RUNX2 (green) and OCN (red) expression with DAPI counterstain (blue). Scale bar, 50 μm.

**Figure S21.** Quantitative analysis of RUNX2 and OCN expression.

**a-b,** Quantitative analysis of RUNX2‑positive (a) and OCN‑positive (b) area from immunofluorescence staining shown in Figure 6d. Data are shown as mean ± SD (n = 3). ***p < 0.001.

**Figure S22.** Immunofluorescence for NF‑κB pathway validation.

**a,** Representative images of p65 (green) and DAPI (blue) in each group. Scale bar, 20 μm.

**b,** Quantitative analysis of p65 fluorescence intensity. Data are shown as mean ± SD (n = 3). **p < 0.01, ***p < 0.001.

**Figure S23.** Immunofluorescence data for Wnt/β‑catenin pathway validation.

**a-c,** Representative images of β‑catenin (a), RUNX2 (b), OCN (c), and DAPI in each group. Scale bar, 20 μm. **d-f,** Quantitative analysis of β‑catenin (d), RUNX2 (e) and OCN (f) fluorescence intensity. Data are shown as mean ± SD (n = 3). ***p < 0.001.

**Figure S24.** Serum biochemical parameters of liver and kidney function.

**a-b,** AST (a) and BUN (b) levels in serum from each group. Data are shown as mean ± SD (n = 3).

**Figure S25.** Quantitative analysis of *in vivo* retention.

Quantitative analysis of *in vivo* fluorescence intensity at the tibial defect over time, representative images were shown in Figure 8f. Data are shown as mean ± SD (n = 3). ***p < 0.001.

**Figure S26.** *Ex vivo* tissue distribution and clearance of SGNCs.

Representative fluorescence images of major organs (heart, liver, spleen, lung, kidney) and tibia at 24 h, 72 h, and 168 h after local administration of Cy5-labeled Sim+GA (upper) or Cy5-labeled SGNCs (lower).

**Table S1.** Sequences of genes selected for analysis by qRT-PCR.

| Gene | Forward primer (5′ to 3′) | Reverse primer (5′ to 3′) |
| --- | --- | --- |
| Gapdh | ACCACCCTGTTGCTGTAGCCAA | GTCTCCTCTGACTTCAACAGCG |
| Tnf | CCAACTCCGGGCTCAGAATT | TCCAGTGAGTTCCGAAAGCC |
| Tgfb1 | CGTTACCTTGGTAACCGGCT | AGCCCTGTATTCCGTCTCCT |
| Runx2 | TGCCATTCGAGGTGGTCG | TCTTCCCAAAGCCAGAGCG |
| Ocn | AGGTAGCGCCGGAGTCTATTCA | GGTGCAGACCTAGCAGACACCA |
